# Development of Polyimides with Low Dielectric Loss Tangent by Incorporating Polysiloxanes with Phenyl Side Groups

**DOI:** 10.1002/marc.202500115

**Published:** 2025-04-07

**Authors:** Riku Takahashi, Ririka Sawada, Kan Hatakeyama‐Sato, Yuta Nabae, Shinji Ando, Teruaki Hayakawa

**Affiliations:** ^1^ Department of Materials Science and Engineering School of Materials and Chemical Technology Institute of Science Tokyo S8‐36 2‐12‐1 Ookayama, Meguro‐ku Tokyo 152‐8552 Japan; ^2^ Department of Chemical Science and Engineering School of Materials and Chemical Technology Institute of Science Tokyo 2‐12‐1 Ookayama, Meguro‐ku Tokyo 152‐8552 Japan

**Keywords:** copolymer, dielectric loss tangent, poly(dimethylsiloxane), poly(diphenylsiloxane), poly[methyl(phenyl)siloxane], polyimide

## Abstract

Owing to their low dielectric constant (*D*_k_), processability, and mechanical properties, siloxane‐based polymers have attracted attention as insulating materials for next‐generation communication. However, a major challenge regarding siloxane‐containing materials is their high dielectric loss tangent (dissipation factor) (*D*_f_). A polymer is designed and synthesized by combining polysiloxanes with phenyl side groups on the main chain and a polyimide structure (polysiloxane‐imide) to improve the *D*_f_ value. Compared with conventional dimethylsiloxane‐based polymers, the resulting polysiloxane‐imide, obtained as a bendable, self‐supporting film, exhibits a significantly reduced *D*_f_ value. The rigidity of the phenyl group‐containing polysiloxane presumably contributes to the improvement in the *D*_f_ value. Furthermore, polysiloxane‐imides exhibit excellent hydrophobicity and high heat resistance with their 5% weight loss temperature of over 400 °C. The synthesized polysiloxane‐imides with phenyl side groups, which possess various properties, including low *D*_k_, low *D*_f_, and excellent hydrophobicity, are expected to contribute to the future practical application of siloxane‐based insulating materials.

## Graphical abstract

Siloxane‐based insulating materials with low dielectric loss tangent (*D*_f_) are developed through the incorporation of polysiloxane with phenyl side groups into polyimide backbone (polysiloxane‐imide). The rigidity of phenyl‐containing polysiloxanes contributes to the reduction of *D*_f_ value. Furthermore, polysiloxane‐imides exhibit high thermal stability with 5% weight loss temperatures of over 400 °C and excellent hydrophobicity.

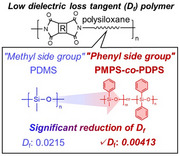

## 1 Introduction

While next‐generation communications utilizing high‐frequency radio waves are expected to be implemented shortly to enable high‐speed and large‐capacity transmission, addressing the transmission loss caused by the insulating materials inside electronic devices, which increases proportionally with the frequency, remains a significant challenge. Considering the transmission loss is proportional to the square root of the dielectric constant (*D*_k_) and dielectric loss tangent (*D*_f_), developing materials with lower *D*_k_ and *D*_f_ values could address this issue.

Aiming to develop insulating materials for next‐generation communications, several studies have attempted to modify the chemical structures of the backbone based on polymers such as polyimide,^[^ ^1^, ^2^, ^3^ ^]^ poly(2,6‐dimethyl‐1,4‐phenylene ether),^[^ ^4^, ^5^ ^]^ polybenzoxazole,^[^ ^6^, ^7^ ^]^ and epoxy resins.^[^ ^8^, ^9^, ^10^ ^]^ A widely studied example of low *D*_k_ and *D*_f_ is the introduction of fluorine structures. The C─F bond possesses a low dipole polarization ability and large volume, thereby enabling improved dielectric properties.^[^ ^11^, ^12^, ^13^, ^14^ ^]^ However, when used in industrial applications, these materials tend to be avoided because of concerns regarding environmental contamination caused by per‐ and polyfluoroalkyl substances (PFAS).^[^ ^15^, ^16^, ^17^ ^]^ Other approaches, such as the introduction of an alicyclic structure,^[^ ^18^, ^19^ ^]^ construction of a porous structure,^[^ ^20^, ^21^, ^22^, ^23^ ^]^ and the addition of a siloxane component^[^ ^24^, ^25^, ^26^, ^27^, ^28^, ^29^ ^]^ have also been applied to develop materials with good dielectric properties. Among these, siloxane‐containing materials stand out owing to their notable characteristics, including thermal stability over a wide temperature range, chemical stability, improved rigidity, processability, and thermal expansion properties,^[^ ^30^, ^31^ ^]^ in addition to a low *D*_k_ value derived from their hydrophobicity and large free volume. As a result, siloxane‐based materials with these properties have attracted growing interest owing to their potential use in bendable and stretchable devices. Previous studies have reported that polyimides containing siloxane units, such as tetramethyldisiloxane,^[^ ^24^ ^]^ hexamethyltrisiloxane,^[^ ^25^ ^]^ poly(dimethylsiloxane),^[^ ^26^, ^32^ ^]^ and cross‐linked siloxane,^[^ ^27^ ^]^ exhibit superior *D*_k_ values compared to ordinary polyimides. However, conventional siloxane‐containing materials have high *D*_f_ values. Dimethylsiloxane units have been used as the siloxane components, with their extremely flexible chains considered to result in undesirable *D*_f_ values. Currently, significantly reducing *D*_f_ values of siloxane‐based insulating polymers has been required.

As a strategy to improve *D*_f_, rigidifying the polymer backbone^[^ ^33^, ^34^, ^35^ ^]^ and introducing rigid or bulky functional groups into the side chains,^[^ ^36^ ^]^ thereby reducing the mobility of the molecular chains, have been reported to be effective. For example, liquid‐crystalline polyimide developed by introducing a planar backbone structure showed an improved *D*_f_ value owing to its oriented main chain and suppressed molecular motion.^[^ ^34^ ^]^ In another study, introducing a biphenyl side group restrained the rotation of the main chain, reducing the *D*_f_ value.^[^ ^36^ ^]^

Regarding the siloxane backbone, poly(diphenylsiloxane) (PDPS), a representative polysiloxane containing phenyl groups, possesses a rigid crystalline structure.^[^ ^37^ ^]^ Thus, adding aromatic side groups instead of methyl groups is expected to be an effective approach for rigidifying the siloxane backbone. However, considering PDPS has extremely low solubility and is difficult to handle,^[^ ^38^, ^39^, ^40^ ^]^ incorporating diphenylsiloxane components through copolymerization or using different siloxane monomer units with phenyl side groups is necessary.

We designed and synthesized polymers by combining the properties of polysiloxanes with phenyl side groups and polyimide. Introducing phenyl groups facilitates the development of rigid siloxane chains, resulting in a lower *D*_f_ value than that of a conventional dimethylsiloxane‐containing polyimide. Additionally, while the polysiloxane itself is a viscous liquid, incorporating polysiloxanes into the polyimide backbone is expected to enable the fabrication of a self‐standing film rich in siloxane components with sufficient strength. As a polysiloxane with phenyl groups, copolymers of poly(dimethylsiloxane) (PDMS) and PDPS (PDMS‐*co*‐PDPS) containing phenyl groups were synthesized. This method aimed to achieve both the overcoming of the poor solubility of PDPS and the imparting of rigidity. Poly [methyl(phenyl)siloxane] (PMPS), easier to handle than PDPS, was employed as another siloxane candidate. To further enhance the rigidity of the siloxane chain, a copolymer of PMPS and PDPS (PMPS‐*co*‐PDPS) was synthesized, and its properties were investigated. Polysiloxanes were then incorporated into polyimide through a polycondensation reaction and thermal imidization after modifying the terminal functional groups. Accordingly, the dielectric and thermal properties, higher‐order structures, and hydrophobicity of the polysiloxane‐based films were investigated.

## 2 Results and Discussion

### 2.1 Syntheses of Hydrosilyl‐Terminated Polysiloxanes

**Scheme 1:**
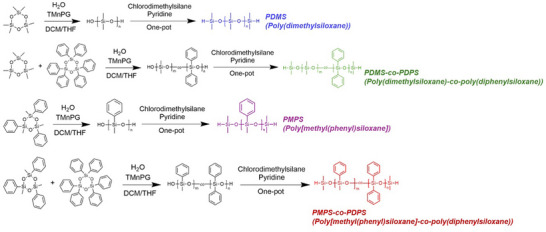
Syntheses of polysiloxanes via ring‐opening polymerization.

Polysiloxanes with and without aromatic groups, such as poly(dimethylsiloxane) (PDMS), poly[methyl(phenyl)siloxane] (PMPS), copolymers composed of PDMS and poly(diphenylsiloxane) (PDPS) (PDMS‐*co*‐PDPS), and a copolymer comprising PMPS and PDPS (PMPS‐*co*‐PDPS) were synthesized via ring‐opening polymerization of cyclotrisiloxane monomers (**Scheme** **1**). 1,3‐Trimethylene‐2‐propylguanidine (TMnPG) and water were used as the organic base catalyst and initiator, respectively. These synthetic methods are based on previous studies of polysiloxane homopolymers^[^ ^38^, ^41^, ^42^, ^43^ ^]^ and copolymers.^[^ ^44^ ^]^ Hydrosilyl groups were introduced at the end of the polymer chain by terminating the polymerization using chlorodimethylsilane as an end‐capping reagent and pyridine as a hydrochloric acid scavenger. The synthesis of polysiloxanes with hydrosilyl end groups was verified by ^1^H‐NMR (Figures S1, S5, S9, S13, S17, and S21, Supporting Information) and size exclusion chromatography (SEC) (Figure S25, Supporting Information). The molecular weights and the polydispersity indices (PDI) of the polysiloxanes employed in this study are listed in **Table** **1**. The subscript numbers on PDMS‐*co*‐PDPS and PMPS‐*co*‐PDPS indicate the weight fractions of the corresponding components estimated by ^1^H‐NMR. The numbers following the names of the polysiloxanes represent their molecular weights. Polysiloxanes with molecular weights of ≈3000 (3k)‐4000 (4k) and 6000 (6k) were synthesized by varying the molar ratio of the initiator to the monomer. The molecular weight distributions of PDMS and PMPS were relatively narrow, similar to those previously reported for controlled polymerization.^[^ ^38^ ^]^ The synthesis of PDMS_0.36_‐*co*‐PDPS_0.64_ 3.4k has been previously reported,^[^ ^44^ ^]^ while in this study, a copolymer possessing a molecular weight of 6000 with approximately the same weight fractions of PDPS was synthesized. PMPS‐*co*‐PDPS was synthesized by the polymerization of a mixture of cyclotrisiloxane monomers. All siloxanes with phenyl side groups showed good solubility in tetrahydrofuran (THF), dichloromethane, and toluene, which were used as solvents in the subsequent conversion of the terminal functional groups and polycondensation reactions.

**Table 1:** Molecular weights (*M*_n_), polydispersity indices (PDI), and glass transition temperatures (*T*_g_) of polysiloxanes.

Polysiloxane	*M*_n, NMR_ [kg mol^−1^]^a)^	*M*_n, SEC_ [kg mol^−1^]^b)^	PDI ^b)^	*T*_g_ [°C]^c)^
PDMS 6.1k	6.1	7.9	1.06	N/A
PMPS 3.3k	3.3	3.0	1.08	−34
PMPS 6.1k	6.1	5.6	1.12	−30
PDMS_0.36_‐*co*‐PDPS_0.64_ 3.4k^d)^	3.4	3.5	1.12	−43
PDMS_0.37_‐*co*‐PDPS_0.63_ 6.0k	6.0	6.3	1.17	−43
PMPS_0.54_‐*co*‐PDPS_0.46_ 4.1k	4.1	3.2	1.18	−7

^a)^ Determined by ^1^H‐NMR of nitro‐terminated polysiloxanes;

^b)^ Determined by SEC of nitro‐terminated polysiloxanes using polystyrene as a standard;

^c)^ Determined by DSC measurement of amino‐terminated polysiloxanes;

^d)^ PDMS_0.36_‐*co*‐PDPS_0.64_ 3.4k was reported in our previous paper.^[^ ^44^ ^]^

### 2.2 Chain‐End Modifications of Polysiloxanes

**Scheme 2:**
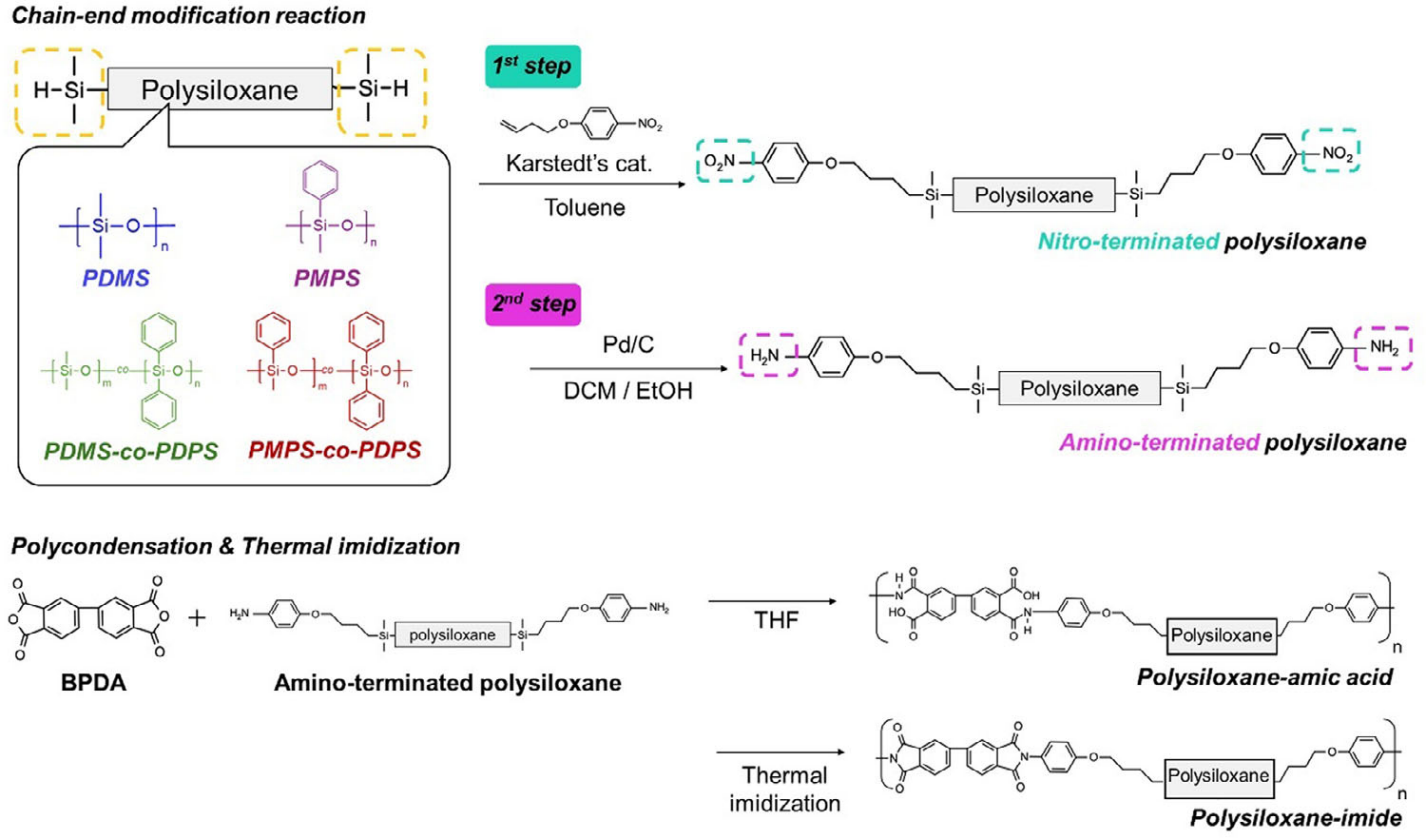
Syntheses of amino‐terminated polysiloxanes and polysiloxane‐imides.

The hydrosilyl groups on the polysiloxane chain ends are converted into amino groups through a two‐step reaction (**Scheme** **2**). First, the end groups were modified with nitro groups via hydrosilylation using Karstedt's catalyst (platinum (0) 1,3‐diethenyl‐1,1,3,3‐tetramethyldisiloxane complexes). The hydrosilyl end‐groups and vinyl groups on 1‐(but‐3‐en‐1‐yloxy)‐4‐nitrobenzene reacted.^[^ ^45^ ^]^ After the reaction, the polysiloxane was purified by column chromatography. The absence of 1‐(but‐3‐en‐1‐yloxy)‐4‐nitrobenzene and the conversion of hydrosilyl groups to nitro groups were verified by ^1^H‐NMR (Figures S2, S6, S10, S14, S18, and S22, Supporting Information). Next, the nitro groups were reduced using Pd/C under an H_2_ atmosphere to obtain polysiloxane with amino groups at the terminals. The conversion of nitro to amino groups was verified by the disappearance of the ^1^H‐NMR peak of the proton at the ortho position relative to the nitro group (Figures S3, S7, S11, S15, S19, and S23, Supporting Information). ^1^H‐NMR peaks for the protons at the ortho‐ and meta‐positions relative to the amino group were also observed after the reaction. Amino‐terminated polysiloxanes were used in the subsequent polycondensation reactions.

### 2.3 Syntheses of Polysiloxane‐Imides

**Figure 1:**
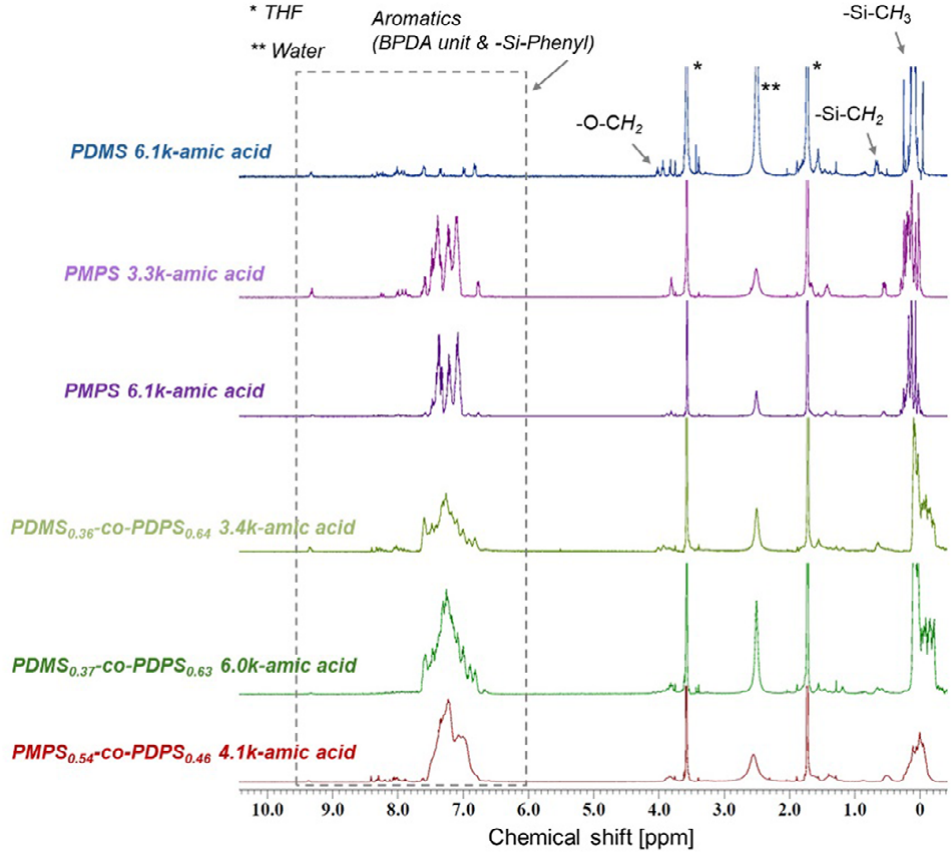
^1^H‐NMR spectra of polysiloxane‐amic acids using THF‐*d*_8_ as a solvent. The ^1^H‐NMR spectrum of PDMS_0.36_‐*co*‐PDPS_0.64_ 3.4k‐amic acid has been reported in our previous study.^[^ ^44^ ^]^

**Figure 2:**
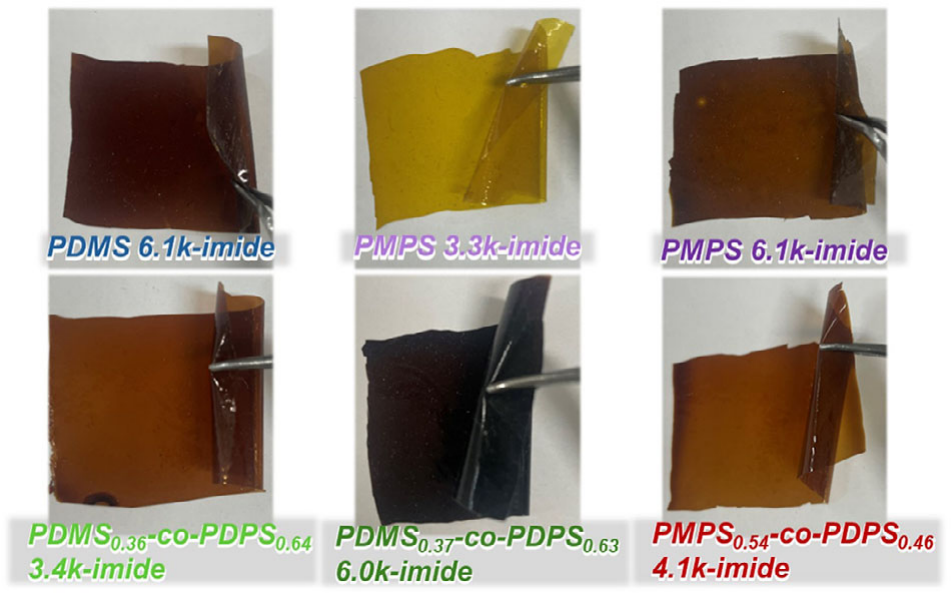
Prepared polysiloxane‐imide films with self‐supporting and bendable properties. Fabrication of PDMS_0.36_‐*co*‐PDPS_0.64_ 3.4k‐imide films has been reported in our previous study.^[^ ^44^ ^]^

**Figure 3:**
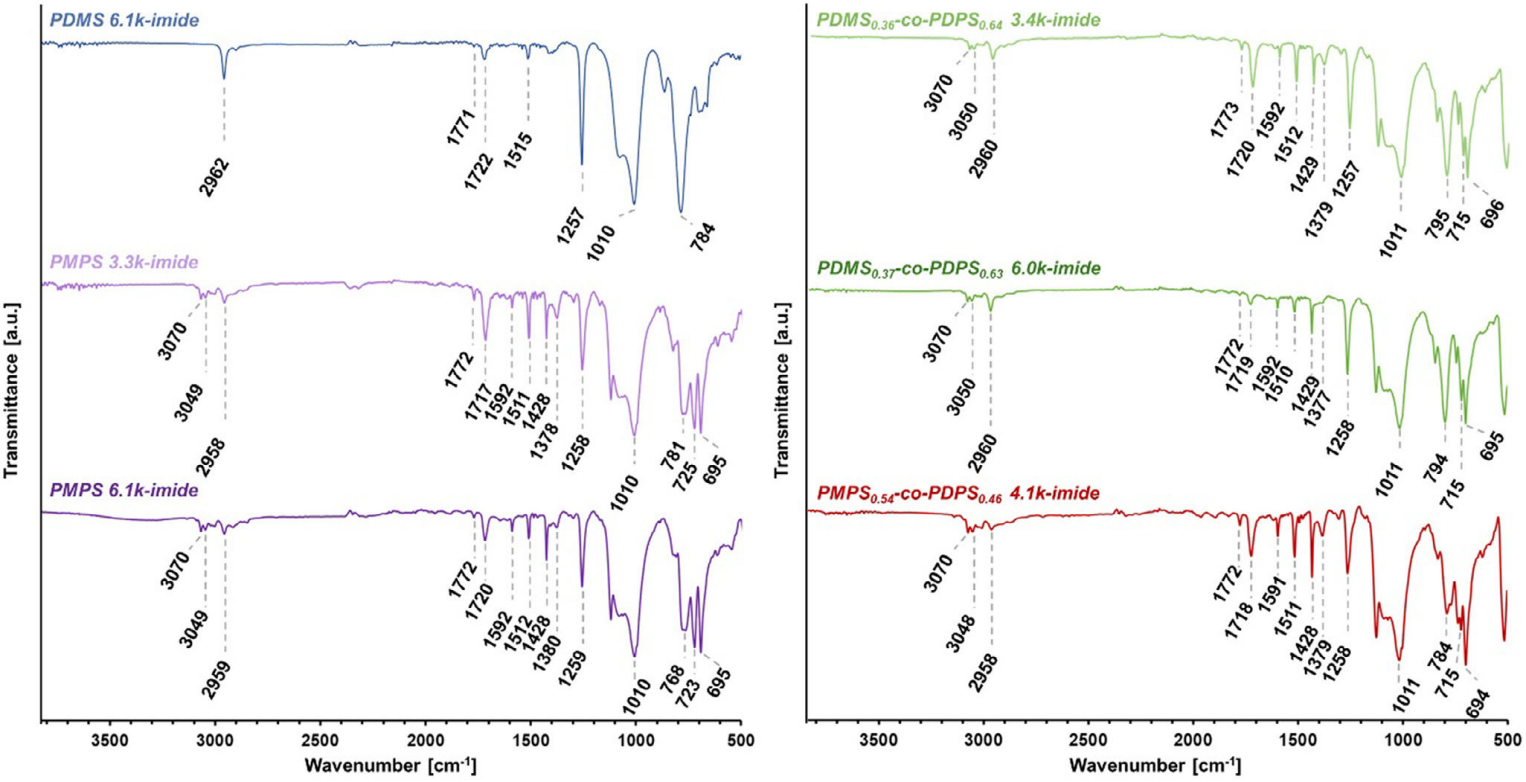
FT–IR spectra of polysiloxane‐imides measured using the attenuated total reflection (ATR) method. FT–IR spectrum of PDMS_0.36_‐*co*‐PDPS_0.64_ 3.4k‐imide has been reported in our previous paper.^[^ ^44^ ^]^ 3070 cm^−1^ (CH aromatic stretching), 3050–3048 cm^−1^ (CH aromatic stretching), 2962–2958 cm^−1^ (CH_3_ stretching), 1773–1771 cm^−1^ (C═O stretching), 1722–1717 cm^−1^ (C═O stretching), 1592–1591 cm^−1^ (C═C aromatic stretching), 1515–1510 cm^−1^ (C═C aromatic stretching), 1429–1428 cm^−1^ (C═C aromatic stretching), 1380–1377 cm^−1^ (CH_3_ bending), 1259–1257 cm^−1^ (Si─CH_3_ bending), 1011–1010 cm^−1^ (Si─O stretching), 795–768 cm^−1^ (Si─C stretching), 725–715 cm^−1^ (CH aromatic bending), and 696–694 cm^−1^ (C═C aromatic bending).

Polysiloxanes were incorporated into a polyimide backbone (polysiloxane‐imide) via polycondensation and thermal imidization. First, polysiloxanes with amic acid structures (polysiloxane‐amic acid) were synthesized by the reaction between amino‐terminated polysiloxanes and 4,4’‐biphthalic anhydride (BPDA).^[^ ^44^, ^46^ ^]^ After the reaction, the solution was reprecipitated in methanol, and polysiloxane‐amic acid was obtained as an elastic solid. The progress of the reaction was confirmed by ^1^H‐NMR (**Figure** **1**; Figures S4, S8, S12, S16, S20, and S24, Supporting Information). SEC analysis of polysiloxane‐amic acids also showed an increase in the molecular weight compared to that of the polysiloxanes before the reaction. The characteristics of the polysiloxane‐amic acids are listed in **Table** **2**. The difference in the molecular weight of polysiloxane‐amic acids presumably arose from using polysiloxane as a macromonomer. The molecular weight of polysiloxane calculated from ^1^H‐NMR is not completely accurate, which could have led to a deviation from the equimolar condition with the BPDA monomer. The deviation is considered to have affected the resulting molecular weight of the polysiloxane‐amic acid. Subsequently, a tetrahydrofuran (THF) solution with a concentration of ≈10 wt.% polysiloxane‐amic acid was cast onto a glass substrate. To obtain polysiloxane‐imide, dried polysiloxane‐amic acid samples on the substrate were subjected to thermal imidization at 100 °C for 1 h, then at 200 °C for 1 h, and finally at 250 or 300 °C for 2 h. Polysiloxane imide was fabricated as a self‐standing film after peeling it off the substrate (**Figure** **2**). The coloration of the films is considered to originate from trace residues of Pd/C. Nitro‐terminated polysiloxanes were colorless and transparent after purification through column chromatography. However, even though Pd/C was removed by filtration, amino‐terminated polysiloxanes exhibited a transparent but brownish color due to a slight amount of residue. The coloration of amino‐terminated polysiloxanes affected the appearance of polysiloxane‐imide films. Fourier transform infrared (FT–IR) spectroscopy was conducted to characterize the polysiloxane‐imides (**Figure** **3**). All the polysiloxane‐imides exhibited peaks at 1773–1771 cm^−1^ and 1722–1717 cm^−1^ derived from an imide structure. Peaks at 1011–1010 cm^−1^, originating from the siloxane backbone (─Si─O─), were also observed for each polysiloxane‐imide. Other distinctive functional groups were detected at 2962–2958 cm^−1^, 1380–1377 cm^−1^, and 1259–1257 cm^−1^ for ─Si─CH_3_ and 3070 cm^−1^, 3050–3048 cm^−1^, 1592–1591 cm^−1^, 1515–1510 cm^−1^, 1429–1428 cm^−1^, and 725–715 cm^−1^ for aromatics.

**Table 2:** Characteristics of polysiloxane‐amic acids or polysiloxane‐imides.

Sample	*M*_w_ [kg mol^−1^]^a)^	Viscosity [dL g^−1^]^b)^	*T*_g_ [°C]^c)^	*T*_d5_ [°C]^d)^	*T*_d10_ [°C]^d)^	*D*_k_ ^e)^	*D*_f_ ^e)^	WAXD diffraction peak [nm]^f)^		Contact angle [°]^g)^
PDMS 6.1k‐amic acid/imide	40.2	0.26	−124	423	446	2.71	0.0215	6.3	0.74	111
PMPS 3.3k‐amic acid/imide	37.8	0.27	−21	401	420	2.85	0.00795	4.4	0.88	102
PMPS 6.1k‐amic acid/imide	61.8	0.27	−24	438	451	2.82	0.00855	13	0.87	107
PDMS_0.36_‐*co*‐PDPS_0.64_ 3.4k‐amic acid/imide^h)^	72.9	0.49	−34	432	446	2.92	0.0108	4.7	0.91	102
PDMS_0.37_‐*co*‐PDPS_0.63_ 6.0k‐amic acid/imide	27.1	0.10	−37	447	466	2.85	0.0129	15	0.90	108
PMPS_0.54_‐*co*‐PDPS_0.46_ 4.1k‐amic acid/imide	25.5	0.20	3.0	414	429	2.91	0.00413	14	0.99	102

^a)^ Weight average molecular weight determined by SEC using polystyrene as a standard;

^b)^ Measured with Ostwald viscometer at 0.5 g dL^−1^ in THF;

^c)^ Glass transition temperature determined by DSC;

^d)^ 5% and 10% weight loss temperature determined by TGA analysis;

^e)^ Dielectric constant and dielectric loss tangent measured at 10 GHz;

^f)^ Diffraction peaks observed in the WAXD profiles;

^g)^ Observed contact angle using water as a droplet;

^h)^ Molecular weight and glass transition temperature of PDMS_0.36_‐*co*‐PDPS_0.64_ 3.4k‐amic acid have been reported in our previous study.^[^ ^44^ ^]^

### 2.4 Thermal Property of Polysiloxane‐Imides

**Figure 4:**
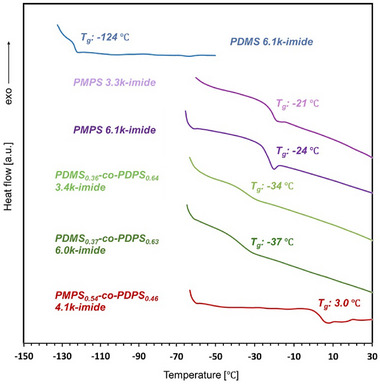
DSC curves of polysiloxane‐imides. Glass transition temperatures (*T*_g_) are also shown in the profiles. The DSC result of PDMS_0.36_‐*co*‐PDPS_0.64_ 3.4k‐imide has been reported in our previous work.^[^ ^44^ ^]^

Differential scanning calorimetry (DSC) and thermogravimetric analysis (TGA) were used to investigate the thermal properties of the polysiloxane‐imides. DSC results measured at a heating rate of 5 °C min^−1^ are shown in **Figure** **4**. Glass transition was observed in all polysiloxane‐imides, with transition temperatures of −124 °C for PDMS‐imide, −24 to −21 °C for PMPS‐imide, −37 to −34 °C for PDMS‐*co*‐PDPS‐imide, and 3.0 °C for PMPS‐*co*‐PDPS‐imide. Compared to the glass transition temperatures (*T*_g_) of the polysiloxanes before imidization (Table 1), a slight increase in *T*_g_ was observed for each polysiloxane‐imide upon incorporation into the polyimide backbone, attributed to stiffening of the polymer chains resulting from the introduction of the rigid BPDA unit. Additionally, the *T*_g_ values observed in PDMS‐*co*‐PDPS and PMPS‐*co*‐PDPS before imidization were consistent with those estimated using the Fox equation (Table S1, Supporting Information).^[^ ^47^ ^]^ Furthermore, despite the *T*_g_ of the synthesized polysiloxane‐imide films being below room temperature, they were all obtained as self‐supporting films, likely attributable to the interactions between the BPDA imide groups introduced via the polycondensation reaction, which led to the formation of a physical crosslinking‐like structure. Similar to this study, the fabrication of polyimide films with main chain backbones exhibiting *T*_g_ values lower than room temperature was reported in a previous study.^[^ ^31^ ^]^

**Figure 5:**
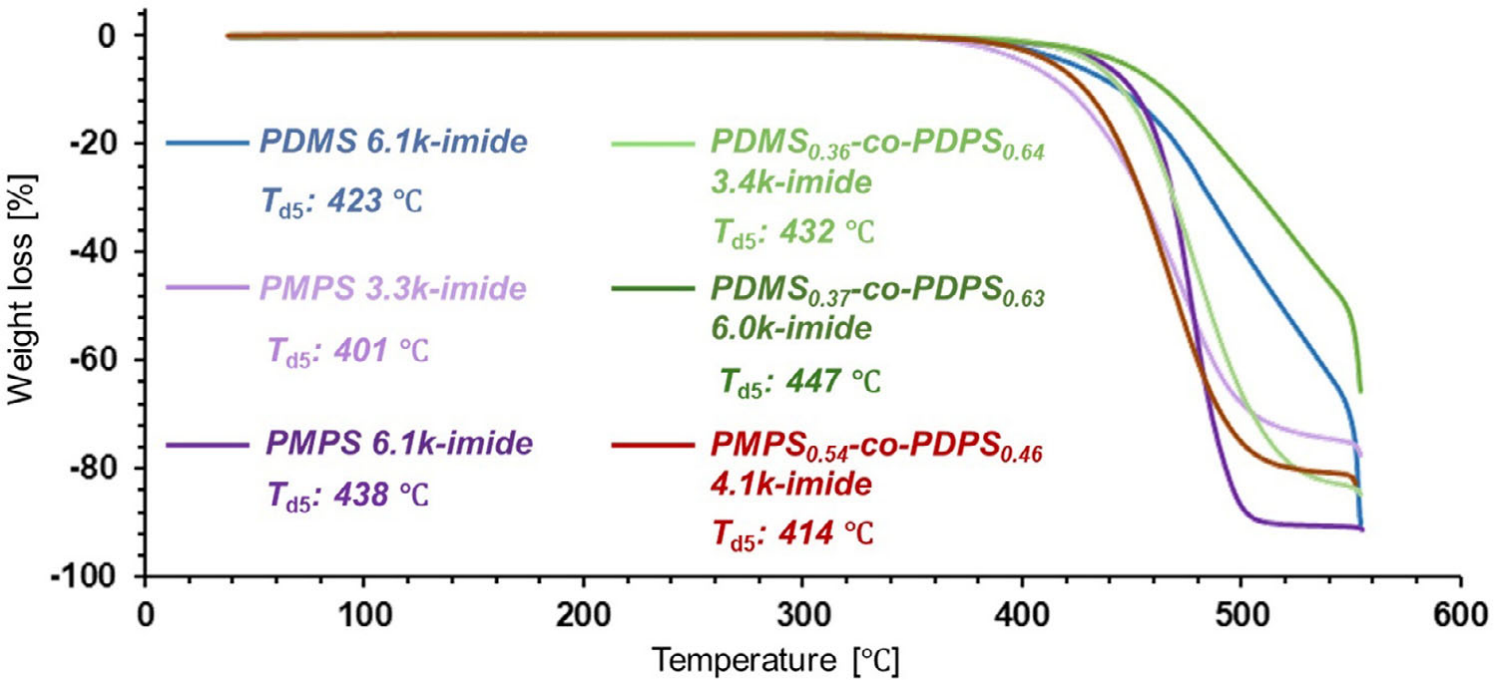
Weight loss of polysiloxane‐imides upon heating under nitrogen atmosphere. 5% weight loss temperatures (*T*_d5_) are also shown in the figure.

Heat resistance is an important property when polysiloxane‐imide films are used as insulating materials in industrial applications. **Figure** **5** shows the results of TGA for polysiloxane‐imides measured at a heating rate of 10 °C min^−1^. All the polysiloxane‐imides exhibited high thermal durability with their 5% weight loss temperature (*T*_d5_) above 400 °C. In particular, PMPS 6.1k‐imide and PDMS_0.37_‐*co*‐PDPS_0.63_ 6.0k‐imide possessed higher *T*_d5_ values than the previously reported polyimide containing dimethylsiloxane structure.^[^ ^31^ ^]^ When comparing the *T*_d5_ values of polysiloxanes before incorporation into the polyimide backbone, polysiloxanes containing aromatic rings (PMPS, PDMS‐*co*‐PDPS, and PMPS‐*co*‐PDPS) showed higher *T*_d5_ values than PDMS (Table S2, Supporting Information). However, a difference in heat resistance depending on the presence or absence of aromatic rings was not observed for the polysiloxane‐imides. In the case of PDMS 6.1k‐imide (*T*_d5_: 423 °C), relative to PDMS homopolymer (*T*_d5_: 323 °C), the heat resistance improved upon the introduction of BPDA imide units, which possess thermal durability. In contrast, for aromatic polysiloxanes, the thermal decomposition temperature decreased after incorporation into the polyimide backbone, attributed to the inferior heat resistance of the ─(CH_2_)_4_─O─ units (added during the chain‐end modification process).

### 2.5 Dielectric Properties of Polysiloxane‐Imides

Dimethylsiloxane, one of the siloxanes with the simplest chemical structure, has been leveraged to improve the dielectric properties of insulating materials such as polyimides. For instance, the introduction of tetramethyldisiloxane,^[^ ^24^ ^]^ hexamethyltrisiloxane,^[^ ^25^ ^]^ and poly(dimethylsiloxane)^[^ ^26^, ^32^ ^]^ have led to a reduction in dielectric constant (*D*_k_). However, incorporating a highly flexible dimethylsiloxane component poses the challenge of increasing the dielectric loss tangent (*D*_f_). To investigate the effects of adding phenyl side groups instead of methyl groups, we measured *D*_k_ and *D*_f_ values.

**Figure 6:**
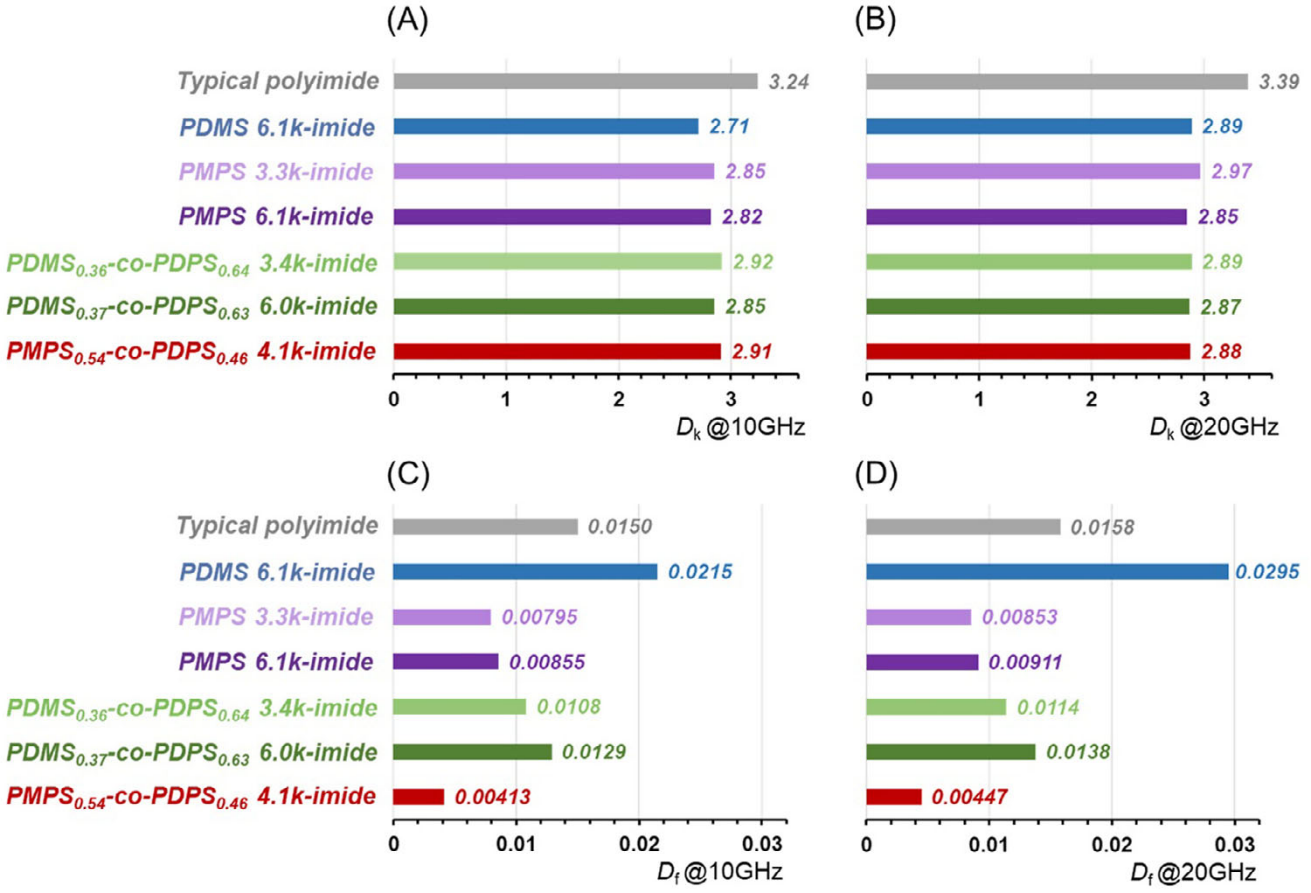
Dielectric constant (*D*_k_) and dielectric loss tangent (*D*_f_) of polysiloxane‐imides measured at 10 and 20 GHz.

The *D*_k_ and *D*_f_ values of the synthesized polysiloxane‐imides are shown in **Figure** **6**. The values for a typical polyimide synthesized from PMDA (pyromellitic dianhydride) and ODA (4,4’‐oxydianiline) are also included for comparison. As observed in previous studies, PDMS 6.1k‐imide showed a lower *D*_k_ than conventional polyimide, but *D*_f_ was significantly higher. Polysiloxane‐imides with aromatic rings in the side groups (PMPS 3.3k‐imide, PMPS 6.1k‐imide, PDMS_0.36_‐*co*‐PDPS_0.64_ 3.4k‐imide, PDMS_0.37_‐*co*‐PDPS_0.63_ 6.0k‐imide, and PMPS_0.54_‐*co*‐PDPS_0.46_ 4.1k‐imide) also exhibited lower *D*_k_ compared to typical polyimides at both 10 and 20 GHz. The effect of introducing phenyl groups in place of the methyl group on the chain side on the *D*_k_ value was minimal. Furthermore, polysiloxane‐imides with phenyl groups can significantly reduce *D*_f_ compared with PDMS 6.1k‐imide. Notably, both low *D*_k_ and *D*_f_ can be achieved simultaneously in these polysiloxane‐imides, compared to typical polyimides. Figure S26 (Supporting Information) shows the relationship between *D*_f_ and the glass transition temperature (*T*_g_) measured by DSC for each polysiloxane‐imide. As *T*_g_ increased and the siloxane chains became more rigid, *D*_f_ decreased, consistent with previous reports suggesting that making polymer chains rigid and less mobile or less rotationally flexible helps obtain a low *D*_f_ material.^[^ ^33^, ^34^, ^35^, ^36^ ^]^ The properties of siloxane‐based polyimides were previously controlled by altering the length and ratio of the dimethylsiloxane units.^[^ ^24^, ^25^, ^26^ ^]^ Polysiloxane‐imides with phenyl side groups developed in this study exhibited lower *D*_f_ values than previously reported dimethylsiloxane‐based polyimides. These results indicate that utilizing polysiloxanes with phenyl side groups is more beneficial than adjusting the composition or the length of the siloxane units when aiming for lower *D*_f_ values. Regarding the small residual fraction of Pd/C in polysiloxane‐imides, which was mentioned in the synthesis section, we consider that the influence of the residues is minimal since the *D*_k_ value of PDMS 6.1k‐imide is comparable to those reported for dimethylsiloxane‐containing polyimides.^[^ ^25^, ^26^ ^]^

Concerning the molecular weight of polysiloxanes and frequency, among the polysiloxane‐imides with the same chemical structure, polysiloxanes with higher molecular weights resulted in enhanced *D*_f_. This trend was observed at both the 10 and 20 GHz frequencies, attributed to the fact that the siloxane component contributes more significantly to the increase in *D*_f_ than the BPDA unit. Therefore, the polysiloxane‐imide with a higher molecular weight of polysiloxane, which possesses a larger weight fraction of siloxane, exhibited a higher *D*_f_. In contrast, *D*_k_ was slightly lower for the polysiloxane‐imide with a larger molecular weight of polysiloxane, presumably owing to the decrease in the proportion of polar imide groups as the weight fraction of siloxane increased. Additionally, the *D*_f_ values at 20 GHz were higher than those at 10 GHz for all samples, although no significant frequency‐dependent differences were observed in *D*_k_ values.

### 2.6 Wide‐Angle X‐Ray Diffraction (WAXD) Analysis of Polysiloxane‐Imides

**Figure 7:**
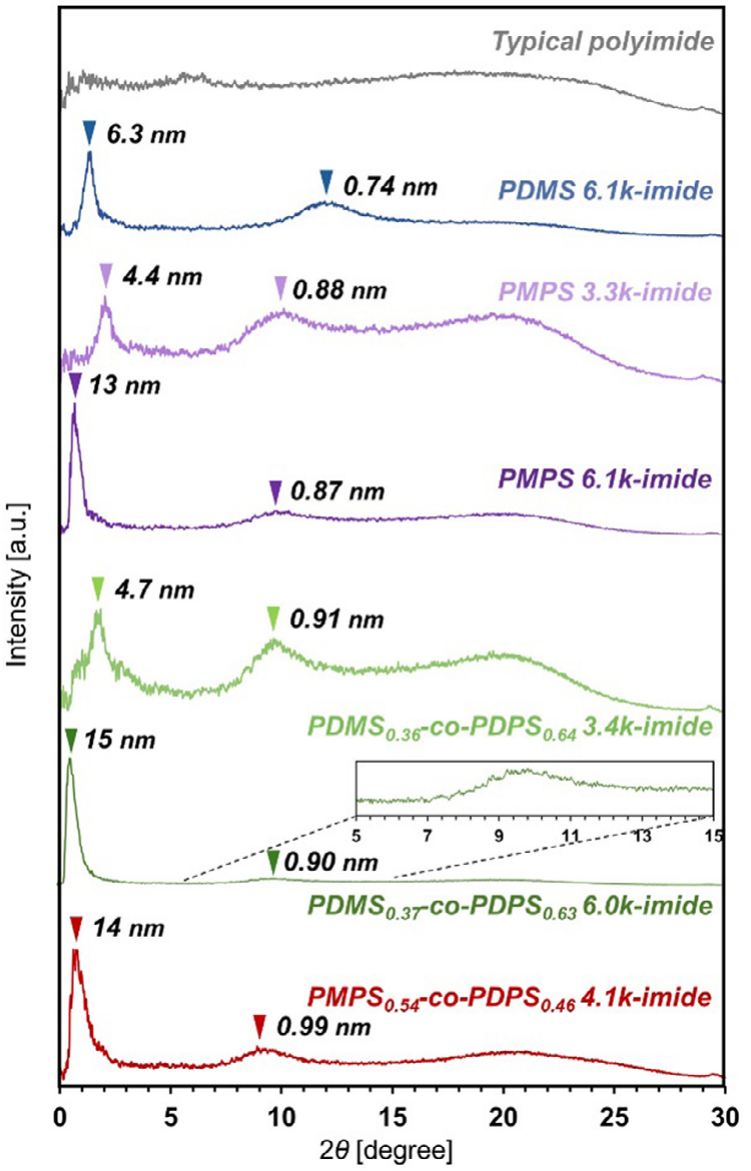
WAXD profiles of polysiloxane‐imides and typical polyimide (PMDA/ODA). The *d*‐spacing of each diffraction peak is displayed near the respective peak. WAXD data of PDMS_0.36_‐*co*‐PDPS_0.64_ 3.4k‐imide has been reported in our previous work.^[^ ^44^ ^]^

Previous studies reported that siloxane‐containing polyimides form ordered structures.^[^ ^31^, ^48^ ^]^ The unique higher‐order structures of the prepared polysiloxane‐imides were analyzed using wide‐angle X‐ray diffraction (WAXD). **Figure** **7** shows the WAXD profiles of a typical polyimide composed of PMDA/ODA monomers and polysiloxane‐imides. The typical polyimide did not form a clearly ordered structure. In contrast, in polysiloxane‐imides, diffraction peaks appeared in around 2*θ* = 0.5°–2.0° and around 10° regions. These peaks correspond to *d*‐spacings of ≈4–15 nm and 0.8 nm, respectively. Additionally, broad amorphous halo diffraction was observed in the wider‐angle region.

First, concerning the smaller‐angle peak with a *d*‐spacing of ≈4–15 nm, in the polysiloxane‐imides composed of PMPS and PDMS‐*co*‐PDPS with different molecular weights, the *d*‐spacing increased as the molecular weight of polysiloxane increased, suggesting that the smaller‐angle peaks correspond to the repeating lengths of the polysiloxane chains. Additionally, as the aromatic ring content in the chemical structure of polysiloxane increased, the *d*‐spacing of the smaller‐angle peak also increased.

For the wider‐angle peak, the polysiloxane‐imides with the same chemical structure exhibited similar *d*‐spacing values, suggesting that the *d*‐spacing of the wider‐angle diffraction peak is attributable to the chemical structure of polysiloxane. These peaks presumably correspond to the distances between polysiloxane chains. Diffraction peaks with similar *d*‐spacing values, estimated to be derived from the intermolecular spacing of the polymer chains, have been observed in siloxane‐containing polyimides.^[^ ^31^, ^48^ ^]^ In addition, as the aromatic ring content in the polysiloxane increased, the *d*‐spacing of the wider‐angle peak also increased, presumed to be due to the bulkiness of the aromatic rings.

The higher‐order structure of the polysiloxane‐imide estimated from WAXD is shown in Figure S27 (Supporting Information). The main chain, composed of polysiloxane and BPDA units, tends to adopt an extended conformation along the direction of the molecular chain. Furthermore, the main chains partially overlapped each other, developing an ordered structure with a *d*‐spacing of ≈0.8 nm.

### 2.7 Hydrophobicity Evaluation of Polysiloxane‐Imide Films

**Figure 8:**
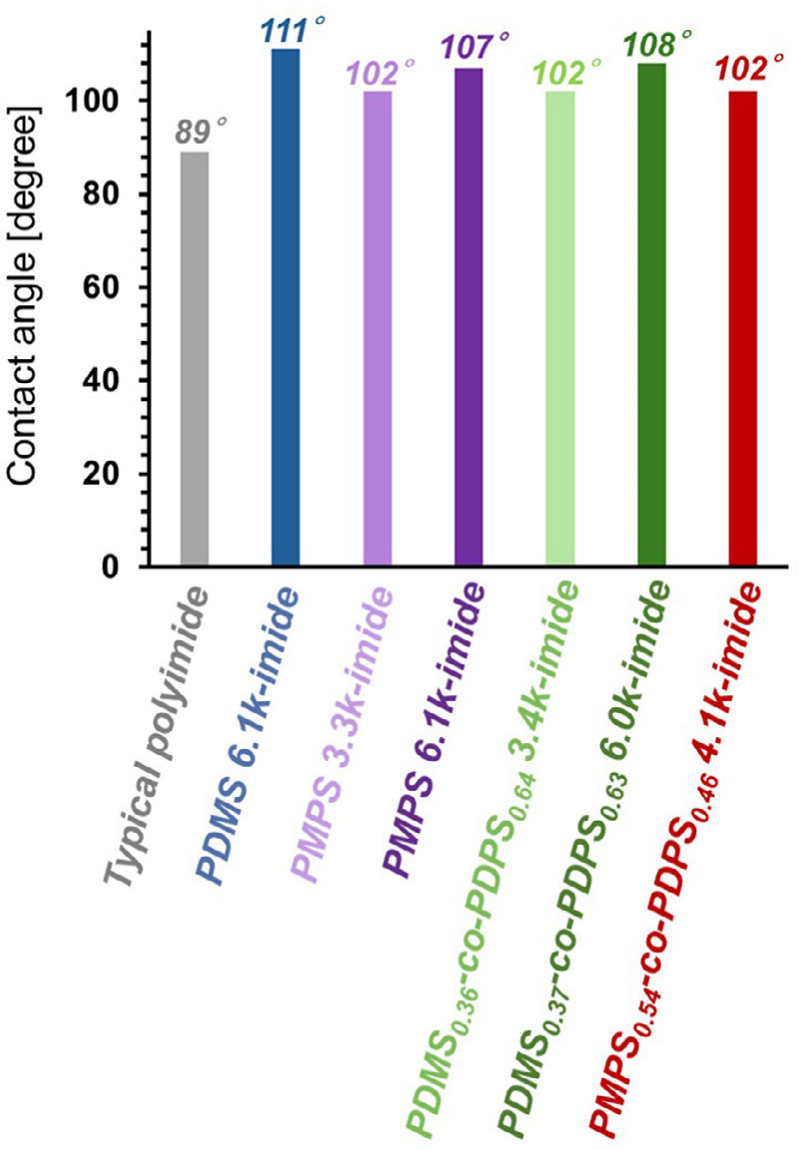
The contact angle of a water droplet on the polysiloxane‐imide films and the typical polyimide film (PMDA/ODA). Snapshots of the contact angle measurements are shown in Figure S28 (Supporting Information).

The hydrophobicity of insulating material is extremely important^[^ ^49^, ^50^ ^]^ because water adsorption and absorption definitely increase the dielectric constant (*D*_k_) and dielectric loss tangent (*D*_f_). Polyimide exhibits higher *D*_k_ and *D*_f_ values under higher humidity conditions, attributed to water adsorbed on the polyimide.^[^ ^51^, ^52^ ^]^ We measured the surface contact angle with water droplets to investigate the hydrophobicity of the polysiloxane‐imide films. The contact angles of typical polyimide composed of PMDA/ODA monomers and polysiloxane‐imides are shown in **Figure** **8**. PDMS 6.1k‐imide exhibited higher hydrophobicity than the typical polyimide, as demonstrated by its larger contact angle, thus validating the higher hydrophobicity previously reported for polyimides containing dimethylsiloxane units.^[^ ^26^ ^]^ Polysiloxane‐imides made from phenyl‐group‐containing polysiloxanes (PMPS, PDMS‐*co*‐PDPS, and PMPS‐*co*‐PDPS) also exhibited enhanced hydrophobicity, similar to that of PDMS 6.1k‐imide. These results revealed that introducing aromatic rings instead of methyl groups did not hinder the hydrophobicity of the polysiloxane‐imides. Thus, it was found that siloxane‐based materials simultaneously achieving low *D*_k_, low *D*_f_, and excellent hydrophobicity were obtained by introducing phenyl side groups. In addition, experimental results indicated that higher hydrophobicity could be achieved by increasing the proportion of the siloxane component, as evidenced by comparing the contact angles of PMPS‐imide and PDMS‐*co*‐PDPS‐imide with polysiloxanes of different molecular weights.

## 3 Conclusion

We synthesized polysiloxane‐imides with phenyl side groups on the siloxane backbone. These polysiloxane‐imides simultaneously achieved lower *D*_k_ and *D*_f_ values than typical polyimides. In particular, PMPS_0.54_‐*co*‐PDPS_0.46_ 4.1k‐imide exhibited a superior *D*_f_ value (*D*_f_ = 0.00413), showing a significant improvement in siloxane‐containing insulating polymers. The excellent *D*_f_ value was presumably due to the enhanced rigidity of the polysiloxane chains caused by the introduction of phenyl side groups. In addition, polysiloxane‐imides possessed good surface hydrophobicity and high thermal stability with their *T*_d5_ of over 400 °C. Notably, the WAXD measurements revealed a unique higher‐order structure resulting from the incorporation of long siloxane chains. We expect that polysiloxane‐imides, which simultaneously possess low *D*_k_, low *D*_f_, and excellent hydrophobicity, will contribute to the development of advanced siloxane‐based insulating materials.

## 4 Experimental Section

### Materials

Hexamethylcyclotrisiloxane (D_3_^(Me,Me)^), pyridine, chlorodimethylsilane, palladium 10% on carbon (wetted with ≈55% water) (Pd/C), 4,4′‐biphthalic anhydride (BPDA), pyromellitic dianhydride (PMDA), 4,4′‐oxydianiline (ODA), 4‐bromo‐1‐butene, 4‐nitrophenol, dichloro(methyl)phenylsilane, 1,3‐diaminopropane, propyl amine, and hexaphenylcyclotrisiloxane (D_3_^(Ph,Ph)^) were purchased from Tokyo Chemical Industry Co., Ltd. Potassium carbonate, carbon disulfide, sodium hydroxide, hydrochloric acid, methyl iodide N,N‐dimethylacetamide (DMAc), DMAc Super dehydrated, and 3A molecular sieves were purchased from FUJIFILM Wako Pure Chemical Corporation. Karstedt's catalyst (Platinum (0) 1,3‐diethenyl‐1,1,3,3‐tetramethyldisiloxane complexes in xylene, Pt ≈2%), celite (Celite 545), and Amberlyst A26 (OH^−^ form) were purchased from Sigma–Aldrich Co. LLC. Dichloromethane was purchased from AGC, Inc. Tetrahydrofuran (THF), ethyl acetate, toluene, hexane, and ethanol were purchased from Godo Co., Ltd. All chemicals were used as received unless otherwise specified. The dried solvents were prepared using molecular sieves as desiccants.^[^ ^53^ ^]^ 1‐(but‐3‐en‐1‐yloxy)‐4‐nitrobenzene,^[^ ^45^, ^46^ ^]^ 1,3‐trimethylene‐2‐propylguanidine (TMnPG),^[^ ^38^ ^]^ and 1,3,5‐trimethyl‐1,3,5‐triphenylcyclotrisiloxane (D_3_^(Me,Ph)^)^[^ ^38^, ^54^ ^]^ were synthesized according to literature methods.

### Measurements

JEOL JNM‐ECS 400 spectrometer at 400 MHz for ^1^H was used for nuclear magnetic resonance (NMR) measurement. A ShodexGPC‐101 system equipped with a ShodexLF‐804 column and a ShodexRI‐501 detector was used for size exclusion chromatography (SEC). Tetrahydrofuran (THF) was used as the eluent. Polystyrene (Shodex STANDARD SM‐105) was used as a standard to determine the molecular weight and polydispersity index (PDI). Ostwald viscometer was used to measure the viscosity of polymers with 0.5 g dL^−1^ THF solution of a sample at 30 °C. A JASCO FT/IR‐4100 Plus spectrophotometer was used to conduct Fourier transform infrared spectroscopy (FT–IR) measurements. The attenuated total reflection (ATR) method was employed using a diamond prism and an ATR PRO ONE (JASCO). An EXSTAR TG/DTA7300 was used for thermogravimetric analysis (TGA). An EXSTAR DSC 7020 was used for differential scanning calorimetry (DSC) measurements. The heating rate was 10 °C min^−1^ for TGA and 5 °C min^−1^ for DSC. A TE‐mode Cavity Resonator (10 and 20 GHz) (AET, Inc.) equipped with a vector network Analyzer MS46122B (ANRITSU Corp.) was used to measure the dielectric constant (*D*_k_) and dielectric loss tangent (*D*_f_). Averaged *D*_k_ and *D*_f_ values obtained from around five measurements were used. Dielectric property measurement was conducted for around 5 cm × 5 cm films under ≈25 °C and 30% RH conditions. Average film thicknesses measured at 10 points on the film were 217 µm for PDMS 6.1k‐imide, 54 µm for PMPS 3.3k‐imide, 101 µm for PMPS 6.1k‐imide, 64 µm for PDMS_0.36_‐*co*‐PDPS_0.64_ 3.4k‐imide, 117 µm for PDMS_0.37_‐*co*‐PDPS_0.63_ 6.0k‐imide, and 28 µm for PDMS_0.54_‐*co*‐PDPS_0.46_ 4.1k‐imide, and these values were used to calculate *D*_k_ and *D*_f_ values. A Bruker Discover D8 equipped with a Cu (1.54 Å) X‐ray source was used to obtain wide‐angle X‐ray diffraction (WAXD) profiles. A contact angle meter DM‐501YH (Kyowa Interface Science Co., Ltd.) was used to measure the surface contact angle, using water as the liquid.

### Syntheses of Polysiloxanes

Polysiloxanes with hydrosilyl end groups were synthesized via the ring‐opening polymerization of cyclotrisiloxane monomers using water as an initiator and 1,3‐trimethylene‐2‐propylguanidine (TMnPG) as a catalyst.^[^ ^38^, ^41^, ^42^, ^43^, ^44^ ^]^ The hydrosilyl groups were introduced using chlorodimethylsilane as an end‐capping reagent. The synthesis procedure of polydimethylsiloxane (PDMS) is shown below. Hexamethylcyclotrisiloxane (D_3_^(Me,Me)^) (2.5 g, 11.3 mmol) was dissolved in 5.25 mL of dried dichloromethane. A mixture of H_2_O (7.4 mL, 0.41 mmol) and dried THF (1.0 mL) was added to the solution. A toluene solution of 1,3‐trimethylene‐2‐propylguanidine (TMnPG) (0.18 mL, 91 mg mL^−1^) containing 16 mg (0.11 mmol) of TMnPG was added to initiate the polymerization. After 80 min of reaction at 30 °C, chlorodimethylsilane (0.45 mL, 4.1 mmol) and pyridine (0.54 mL, 6.6 mmol) were added to terminate the reaction. The end‐capping reaction was allowed to proceed for 24 h. The mixture was concentrated and washed with acetonitrile. Drying under a vacuum afforded hydrosilyl‐terminated PDMS 6.1k with a yield of 1.71 g (68%). The other polysiloxanes were synthesized by the same procedure using the corresponding cyclotrisiloxane monomers, as shown in the Supporting Information.

### Chain‐End Modifications of Polysiloxanes

Terminal hydrosilyl groups were converted to amino groups via a two‐step reaction. First, nitro groups were introduced via the reaction between hydrosilyl‐terminated polysiloxanes and 1‐(but‐3‐en‐1‐yloxy)‐4‐nitrobenzene using Karstedt's catalyst. The terminal nitro groups were converted into amino groups via catalytic reduction using Pd/C. The specific procedure for PDMS 6.1 K is shown below as an example. The first reaction was conducted using hydrosilyl‐terminated PDMS 6.1k 1.71 g (0.29 mmol), 1‐(but‐3‐en‐1‐yloxy)‐4‐nitrobenzene 0.22 g (1.2 mmol), and dry toluene (9 mL). These chemicals were then added to a test tube. After adding nine drops of Karstedt's catalyst, the reaction was carried out for ≈12 h at 25 °C. The progress of the reaction was verified by ^1^H‐NMR of an aliquot of the reaction mixture. The mixture was concentrated and purified by column chromatography using hexane/dichloromethane (= 5/4) as the eluent. Drying under a vacuum afforded nitro‐terminated PDMS 6.1k with a yield of 1.40 g (80%). In the second reaction, nitro‐terminated PDMS 6.1k 1.40 g, ethanol (4 mL), dichloromethane (4 mL), and Pd/C (70 mg) were added to a flask. The mixture was stirred at room temperature (around 25 °C) under an H_2_ atmosphere using an H_2_ balloon. The ^1^H‐NMR spectrum of an aliquot of the reaction mixture confirmed the complete conversion of the nitro groups. After the reaction, Pd/C was filtered through Celite, and the solution was concentrated. After drying under vacuum, 1.30 g (92%) of amino‐terminated PDMS 6.1k was obtained. These reactions were conducted for all polysiloxanes in the same manner, as shown in the Supporting Information.

### Syntheses of Polysiloxane‐Imides Through the Incorporation of Polysiloxanes into Polyimide Backbone

Amino‐terminated polysiloxanes were introduced into the polyimide backbone via polycondensation reaction with 4,4′‐biphthalic anhydride (BPDA) and thermal imidization. The polymer obtained from the polycondensation reaction was named polysiloxane‐amic acid. The synthesis procedure for PDMS 6.1k‐amic acid is as follows. Amino‐terminated PDMS 6.1k (1.29 g, 0.21 mmol) and THF (4 mL, distilled and dried) were added to a flask. Next, 4,4’‐biphthalic anhydride (BPDA) (62 mg, 0.21 mmol) and THF (2.4 mL, distilled and dried) were added to the solution. The resulting solution was stirred for 24 h under reflux. After the reaction, the solution was reprecipitated in methanol, and the remaining solid was dried under vacuum, ultimately yielding 0.79 g (58%) of PDMS 6.1k‐amic acid. The same procedure was used to synthesize the other polysiloxane‐amic acids, as explained in the Supporting Information. Subsequently, a 10 wt.% THF solution of polysiloxane‐amic acid was cast onto a glass substrate. The cast film was dried under vacuum and subjected to thermal imidization at 100 °C for 1 h, 200 °C for 1 h, and 250 or 300 °C for 2 h. After peeling the film off the substrate, polysiloxanes incorporated into the polyimide structure (referred to as polysiloxane‐imide) were obtained.

## Conflict of Interest

The authors declare no conflict of interest.

## Supporting information



Supporting Information

## Data Availability

The data that support the findings of this study are available in the supplementary material of this article.
