# Supplementary material for: Development of Polyimides with Low Dielectric Loss Tangent by Incorporating Polysiloxanes with Phenyl Side Groups
Source: Macromol Rapid Commun. 2025 Apr 7;46(12):2500115. doi: 10.1002/marc.202500115 (PMC12183146; doi:10.1002/marc.202500115)
Supplement: Supplementary file 1 — Supporting Information [file MARC-46-2500115-s001.docx]

**Development of Polyimides with Low Dielectric Loss Tangent by Incorporating Polysiloxanes with Phenyl Side Groups**

Riku Takahashi^1^, Ririka Sawada^2^, Kan Hatakeyama-Sato^1^, Yuta Nabae^1^, Shinji Ando^2^, Teruaki Hayakawa^1^*

^1^Department of Materials Science and Engineering, School of Materials and Chemical Technology, Institute of Science Tokyo, S8-36 2-12-1 Ookayama, Meguro-ku, Tokyo 152-8552, Japan

^2^Department of Chemical Science and Engineering, School of Materials and Chemical Technology, Institute of Science Tokyo, 2-12-1 Ookayama, Meguro-ku, Tokyo 152-8552, Japan

E-mail: [hayakawa.t.ac@m.titech.ac.jp](mailto:hayakawa.t.ac@m.titech.ac.jp)

Contents

[Syntheses. 3](#_Toc194608952)

[1. Synthesis of PDMS (Poly(dimethylsiloxane)) 6.1k 3](#_Toc194608953)

[1.1. Hydrosilyl-terminated PDMS 6.1k 3](#_Toc194608954)

[1.2. Nitro-terminated PDMS 6.1k 3](#_Toc194608955)

[1.3. Amino-terminated PDMS 6.1k 3](#_Toc194608956)

[1.4. PDMS 6.1k-amic acid (Poly(amic acid) containing PDMS 6.1k) 3](#_Toc194608957)

[2. Synthesis of PMPS (Poly[methyl(phenyl)siloxane]) 3.3k 6](#_Toc194608958)

[2.1. Hydrosilyl-terminated PMPS 3.3k 6](#_Toc194608959)

[2.2. Nitro-terminated PMPS 3.3k 6](#_Toc194608960)

[2.3. Amino-terminated PMPS 3.3k 6](#_Toc194608961)

[2.4. PMPS 3.3k-amic acid (Poly(amic acid) containing PMPS 3.3k) 6](#_Toc194608962)

[Synthesis of PMPS 6.1k 9](#_Toc194608963)

[3.1. Hydrosilyl-terminated PMPS 6.1k 9](#_Toc194608964)

[3.2. Nitro-terminated PMPS 6.1k 9](#_Toc194608965)

[3.3. Amino-terminated PMPS 6.1k 9](#_Toc194608966)

[3.4. PMPS 6.1k-amic acid (Poly(amic acid) containing PMPS 6.1k) 9](#_Toc194608967)

[Synthesis of PDMS_0.36_-*co*-PDPS_0.64_ 3.4k 12](#_Toc194608968)

[4.1. Hydrosilyl-terminated PDMS_0.36_-co-PDPS_0.64_ 3.4k 12](#_Toc194608969)

[4.2. Nitro-terminated PDMS_0.36_-co-PDPS_0.64_ 3.4k 12](#_Toc194608970)

[4.3. Amino-terminated PDMS_0.36_-co-PDPS_0.64_ 3.4k 12](#_Toc194608971)

[4.4. PDMS_0.36_-co-PDPS_0.64_ 3.4k-amic acid (Poly(amic acid) containing PDMS_0.36_-co-PDPS_0.64_ 3.4k) 12](#_Toc194608972)

[Synthesis of PDMS_0.37_-*co*-PDPS_0.63_ 6.0k 16](#_Toc194608973)

[5.1. Hydrosilyl-terminated PDMS_0.37_-co-PDPS_0.63_ 6.0k 16](#_Toc194608974)

[5.2. Nitro-terminated PDMS_0.37_-co-PDPS_0.63_ 6.0k 16](#_Toc194608975)

[5.3. Amino-terminated PDMS_0.37_-co-PDPS_0.63_ 6.0k 16](#_Toc194608976)

[5.4. PDMS_0.37_-co-PDPS_0.63_ 6.0k-amic acid (Poly(amic acid) containing PDMS_0.37_-co-PDPS_0.63_ 6.0k) 16](#_Toc194608977)

[Synthesis of PMPS_0.54_-*co*-PDPS_0.46_.4.1k 20](#_Toc194608978)

[6.1. Hydrosilyl-terminated PMPS_0.54_-co-PDPS_0.46_.4.1k 20](#_Toc194608979)

[6.2. Nitro-terminated PMPS_0.54_-co-PDPS_0.46_.4.1k 20](#_Toc194608980)

[6.3. Amino-terminated PMPS_0.54_-co-PDPS_0.46_.4.1k 20](#_Toc194608981)

[6.4. PMPS_0.54_-co-PDPS_0.46_.4.1k-amic acid (Poly(amic acid) containing PMPS_0.54_-co-PDPS_0.46_.4.1k) 20](#_Toc194608982)

[Synthesis of polyimide composed of PMDA/ODA monomers. 25](#_Toc194608983)

[Thermal analyses. 26](#_Toc194608984)

[Glass transition temperatures estimated using Fox equation. 26](#_Toc194608985)

[TGA results of polysiloxanes. 26](#_Toc194608986)

[Dielectric properties of polysiloxane-imides. 27](#_Toc194608987)

[Relationship between dielectric loss tangent (*D*_f_) and glass transition temperature (*T*_g_). 27](#_Toc194608988)

[WAXD analysis of polysiloxane-imides. 28](#_Toc194608989)

[The schematic image of polysiloxane-imide demonstrated by WAXD measurements. 28](#_Toc194608990)

[Hydrophobicity evaluation of polysiloxane-imide films. 28](#_Toc194608991)

[Snapshots of contact angle measurements. 28](#_Toc194608992)

[Calculation of molecular weight based on ^1^H-NMR spectrum. 29](#_Toc194608993)

[References. 30](#_Toc194608994)

# Syntheses.

## 1. Synthesis of PDMS (Poly(dimethylsiloxane)) 6.1k

### 1.1. Hydrosilyl-terminated PDMS 6.1k

Explained in the article.

### 1.2. Nitro-terminated PDMS 6.1k

Explained in the article.

### 1.3. Amino-terminated PDMS 6.1k

Explained in the article.

### 1.4. PDMS 6.1k-amic acid (Poly(amic acid) containing PDMS 6.1k)

Explained in the article.

Figure S2. ^1^H-NMR spectrum of nitro-terminated PDMS 6.1k (Solvent: CDCl_3_).

Figure S1. ^1^H-NMR spectrum of hydrosilyl-terminated PDMS 6.1k (Solvent: CDCl_3_).

Figure S4. ^1^H-NMR spectrum of PDMS 6.1k-amic acid (Solvent: THF-*d*_8_).

**Figure S3.** ^1^H-NMR spectrum of amino-terminated PDMS 6.1k (Solvent: CDCl_3_).

## 2. Synthesis of PMPS (Poly[methyl(phenyl)siloxane]) 3.3k

### 2.1. Hydrosilyl-terminated PMPS 3.3k

1,3,5-Trimethyl-1,3,5-triphenylcyclotrisiloxane (D_3_^(Me,Ph)^) 4.50 g (11.0 mmol) was dissolved in dried dichloromethane 4.41 mL. Into the solution, a mixture of H_2_O 29 mL (1.6 mmol) and dried THF 3.33 mL was added. A toluene solution of 1,3-trimethylene-2-propylguanidine (TMnPG) (91 mg / mL) 0.17 mL (containing 16 mg (0.11 mmol) of TMnPG) was added to initiate the polymerization. After 30 minutes of reaction at 30 ℃, chlorodimethylsilane 0.86 mL (7.9 mmol) and pyridine 1.03 mL (12.6 mmol) were added to terminate the reaction. The end-capping reaction was carried out for over 24 h. The mixture was concentrated and washed with acetonitrile. Drying under a vacuum afforded hydrosilyl-terminated PMPS 3.3k with a yield of 1.63 g (36 %).

### 2.2. Nitro-terminated PMPS 3.3k

Hydrosilyl-terminated PMPS 3.3k 1.63 g (0.56 mmol), 1-(but-3-en-1-yloxy)-4-nitrobenzene 0.65 g (3.4 mmol), and dried toluene 8 mL were added to a test tube. After adding 18 drops of Karstedt’s cat., the reaction was carried out for about 24 h at 25 ℃. The progress of the reaction was verified by ^1^H-NMR of an aliquot of the reaction mixture. The mixture was concentrated and purified by column chromatography using hexane/ethyl acetate (=10/1) as an eluent. Drying under a vacuum afforded nitro-terminated PMPS 3.3k with a yield of 1.49 g (86 %).

### 2.3. Amino-terminated PMPS 3.3k

Nitro-terminated PMPS 3.3k 1.49 g, ethanol 6 mL, dichloromethane 3 mL, and Pd/C 75 mg were added to a flask. The mixture was stirred at room temperature under H_2_ atmosphere using H_2_ balloon. From the ^1^H-NMR spectrum of an aliquot of the reaction mixture, the complete conversion of nitro groups was verified. After the reaction, Pd/C was filtered through celite, and the solution was concentrated. After drying under vacuum, amino-terminated PMPS 3.3k was obtained with a yield of 0.67 g (45 %).

### 2.4. PMPS 3.3k-amic acid (Poly(amic acid) containing PMPS 3.3k)

Amino-terminated PMPS 3.3k 0.67 g (0.20 mmol) and THF (distilled and dried) 2 mL were added to a flask. To the solution, 4,4’-biphthalic anhydride (BPDA) 60 mg (0.20 mmol) and THF (distilled and dried) 1.4 mL were added. The solution was stirred for over 24 h under a reflux condition. After the reaction, the solution was reprecipitated into methanol, and the remaining solid was dried under a vacuum. PMPS 3.3k-amic acid was obtained with a yield of 0.62 g (85 %).

**Figure S5.** ^1^H-NMR spectrum of hydrosilyl-terminated PMPS 3.3k (Solvent: CDCl_3_).

**Figure S6.** ^1^H-NMR spectrum of nitro-terminated PMPS 3.3k (Solvent: CDCl_3_).

**Figure S7.** ^1^H-NMR spectrum of amino-terminated PMPS 3.3k (Solvent: CDCl_3_).

Figure S8. ^1^H-NMR spectrum of PMPS 3.3k-amic acid (Solvent: THF-*d*_8_).

## Synthesis of PMPS 6.1k

### 3.1. Hydrosilyl-terminated PMPS 6.1k

1,3,5-Trimethyl-1,3,5-triphenylcyclotrisiloxane (D_3_^(Me,Ph)^) 4.50 g (11.0 mmol) was dissolved in dried dichloromethane 5.80 mL. Into the solution, a mixture of H_2_O 14 mL (0.75 mmol) and dried THF 1.94 mL was added. A toluene solution of 1,3-trimethylene-2-propylguanidine (TMnPG) (91 mg / mL) 0.17 mL (containing 16 mg (0.11 mmol) of TMnPG) was added to initiate the polymerization. After 25 minutes of reaction at 30 ℃, chlorodimethylsilane 0.82 mL (7.5 mmol) and pyridine 0.98 mL (12 mmol) were added to terminate the reaction. The end-capping reaction was carried out for over 24 h. The mixture was concentrated and washed with acetonitrile. Drying under a vacuum afforded hydrosilyl-terminated PMPS 6.1k with a yield of 3.70 g (82 %).

### 3.2. Nitro-terminated PMPS 6.1k

Hydrosilyl-terminated PMPS 6.1k 2.0 g (0.33 mmol), 1-(but-3-en-1-yloxy)-4-nitrobenzene 0.26 g (1.3 mmol), and dried toluene 10 mL were added to a test tube. After adding 10 drops of Karstedt’s cat., the reaction was carried out for about 24 h at 25 ℃. The progress of the reaction was verified by ^1^H-NMR of an aliquot of the reaction mixture. The mixture was concentrated and purified by column chromatography using hexane/ethylacetate (=10/1) as an eluent. Drying under a vacuum afforded nitro-terminated PMPS 6.1k with a yield of 1.94 g (94 %).

### 3.3. Amino-terminated PMPS 6.1k

Nitro-terminated PMPS 6.1k 1.94 g, ethanol 6 mL, dichloromethane 4 mL, and Pd/C 0.10 g were added to a flask. The mixture was stirred at room temperature under H_2_ atmosphere using H_2_ balloon. From the ^1^H-NMR spectrum of an aliquot of the reaction mixture, the complete conversion of nitro groups was verified. After the reaction, Pd/C was filtered through celite, and the solution was concentrated. After drying under vacuum, amino-terminated PMPS 6.1k was obtained with a yield of 1.80 g (93 %).

### 3.4. PMPS 6.1k-amic acid (Poly(amic acid) containing PMPS 6.1k)

Amino-terminated PMPS 6.1k 1.79 g (0.29 mmol) and THF (distilled and dried) 6 mL were added to a flask. To the solution, 4,4’-biphthalic anhydride (BPDA) 85 mg (0.29 mmol) and THF (distilled and dried) 3.0 mL were added. The solution was stirred for over 24 h under a reflux condition. After the reaction, the solution was reprecipitated into methanol, and the remaining solid was dried under a vacuum. PMPS 6.1k-amic acid was obtained with a yield of 1.31 g (70 %).

Figure S9. ^1^H-NMR spectrum of hydrosilyl-terminated PMPS 6.1k (Solvent: CDCl_3_).

Figure S10. ^1^H-NMR spectrum of nitro-terminated PMPS 6.1k (Solvent: CDCl_3_).

Figure S12. ^1^H-NMR spectrum of PMPS 6.1k-amic acid (Solvent: THF-*d*_8_).

Figure S11. ^1^H-NMR spectrum of amino-terminated PMPS 6.1k (Solvent: CDCl_3_).

## Synthesis of PDMS_0.36_-*co*-PDPS_0.64_ 3.4k

### 4.1. Hydrosilyl-terminated PDMS_0.36_-co-PDPS_0.64_ 3.4k

Hexamethylcyclotrisiloxane (D_3_^(Me,Me)^) 1.50 g (6.75 mmol) and hexaphenylcyclotrisiloxane (D_3_^(Ph,Ph)^) 1.50 g (2.52 mmol) were dissolved in dried dichloromethane 1.34 mL. Into the solution, a mixture of H_2_O 18 mL (1.0 mmol) and dried THF 0.83 mL was added. 1,3-trimethylene-2-propylguanidine (TMnPG) 13 mg (0.093 mmol) dissolved in dried dichloromethane 3.0 mL was added to initiate the polymerization. After 5 h of reaction at 30 ℃, chlorodimethylsilane 1.10 mL (10.0 mmol) and pyridine 1.32 mL (16.0 mmol) were added to terminate the reaction. The end-capping reaction was carried out for over 24 h. The mixture was concentrated and washed with acetonitrile. Drying under a vacuum afforded hydrosilyl-terminated PDMS_0.36_-*co*-PDPS_0.64_ 3.4k with a yield of 1.06 g (35 %).

### 4.2. Nitro-terminated PDMS_0.36_-co-PDPS_0.64_ 3.4k

Hydrosilyl-terminated PDMS_0.36_-*co*-PDPS_0.64_ 3.4k 1.06 g (0.33 mmol), 1-(but-3-en-1-yloxy)-4-nitrobenzene 0.26 g (1.3 mmol), and dried toluene 5 mL were added to a test tube. After adding 10 drops of Karstedt’s cat., the reaction was carried out for about 24 h at 25 ℃. The progress of the reaction was verified by ^1^H-NMR of an aliquot of the reaction mixture. The mixture was concentrated and purified by column chromatography using hexane/dichloromethane (=5/2) as an eluent. Drying under a vacuum afforded nitro-terminated PDMS_0.36_-*co*-PDPS_0.64_ 3.4k with a yield of 0.71 g (63 %).

### 4.3. Amino-terminated PDMS_0.36_-co-PDPS_0.64_ 3.4k

Nitro-terminated PDMS_0.36_-*co*-PDPS_0.64_ 3.4k 1.72 g, ethanol 7 mL, dichloromethane 3 mL, and Pd/C 0.08 g were added to a flask. The mixture was stirred at room temperature under H_2_ atmosphere using H_2_ balloon. From the ^1^H-NMR spectrum of an aliquot of the reaction mixture, the complete conversion of nitro groups was verified. After the reaction, Pd/C was filtered through celite, and the solution was concentrated. After drying under vacuum, amino-terminated PDMS_0.36_-*co*-PDPS_0.64_ 3.4k was obtained with a yield of 1.49 g (91 %).

### 4.4. PDMS_0.36_-co-PDPS_0.64_ 3.4k-amic acid (Poly(amic acid) containing PDMS_0.36_-co-PDPS_0.64_ 3.4k)

Amino-terminated PDMS_0.36_-*co*-PDPS_0.64_ 3.4k 0.63 g (0.19 mmol) and THF (distilled and dried) 2 mL were added to a flask. To the solution, 4,4’-biphthalic anhydride (BPDA) 55 mg (0.19 mmol) and THF (distilled and dried) 1.2 mL were added. The solution was stirred for over 24 h under a reflux condition. After the reaction, the solution was reprecipitated into methanol, and the remaining solid was dried under a vacuum. PDMS_0.36_-*co*-PDPS_0.64_ 3.4k-amic acid was obtained with a yield of 0.59 g (86 %).

Figure S14. ^1^H-NMR spectrum of nitro-terminated PDMS_0.36_-*co-*PDPS_0.64_ 3.4k (Solvent: CDCl_3_).

Figure S13. ^1^H-NMR spectrum of hydrosilyl-terminated PDMS_0.36_-*co-*PDPS_0.64_ 3.4k (Solvent: CDCl_3_).

Figure S15. ^1^H-NMR spectrum of amino-terminated PDMS_0.36_-*co-*PDPS_0.64_ 3.4k (Solvent: CDCl_3_).

Figure S16. ^1^H-NMR spectrum of PDMS_0.36_-*co-*PDPS_0.64_ 3.4k -amic acid (Solvent: THF-*d*_8_).

## Synthesis of PDMS_0.37_-*co*-PDPS_0.63_ 6.0k

### 5.1. Hydrosilyl-terminated PDMS_0.37_-co-PDPS_0.63_ 6.0k

Hexamethylcyclotrisiloxane (D_3_^(Me,Me)^) 1.50 g (6.75 mmol) and hexaphenylcyclotrisiloxane (D_3_^(Ph,Ph)^) 1.50 g (2.52 mmol) were dissolved in dried dichloromethane 3.0 mL. Into the solution, a mixture of H_2_O 11 mL (0.60 mmol) and dried THF 0.47 mL was added. 1,3-trimethylene--propylguanidine (TMnPG) 13 mg (0.093 mmol) dissolved in dried dichloromethane 1.7 mL was added to initiate the polymerization. After 4 h of reaction at 30 ℃, chlorodimethylsilane 0.66 mL (6.0 mmol) and pyridine 0.78 mL (9.6 mmol) were added to terminate the reaction. The end-capping reaction was carried out for over 24 h. The mixture was concentrated and washed with acetonitrile. Drying under a vacuum afforded hydrosilyl-terminated PDMS_0.37_-*co*-PDPS_0.63_ 6.0k with a yield of 2.41 g (80 %).

### 5.2. Nitro-terminated PDMS_0.37_-co-PDPS_0.63_ 6.0k

Hydrosilyl-terminated PDMS_0.37_-*co*-PDPS_0.63_ 6.0k 2.41 g (0.44 mmol), 1-(but-3-en-1-yloxy)-4-nitrobenzene 0.42 g (2.2 mmol), and dried toluene 12 mL were added to a test tube. After adding 18 drops of Karstedt’s cat., the reaction was carried out for about 24 h at 25 ℃. The progress of the reaction was verified by ^1^H-NMR of an aliquot of the reaction mixture. The mixture was concentrated and purified by column chromatography using hexane/dichloromethane (=5/2) as an eluent. Drying under a vacuum afforded nitro-terminated PDMS_0.37_-*co*-PDPS_0.63_ 6.0k with a yield of 1.14 g (46 %).

### 5.3. Amino-terminated PDMS_0.37_-co-PDPS_0.63_ 6.0k

Nitro-terminated PDMS_0.37_-*co*-PDPS_0.63_ 6.0k 1.14 g, ethanol 4 mL, dichloromethane 3 mL, and Pd/C 0.06 g were added to a flask. The mixture was stirred at room temperature under H_2_ atmosphere using H_2_ balloon. From the ^1^H-NMR spectrum of an aliquot of the reaction mixture, the complete conversion of nitro groups was verified. After the reaction, Pd/C was filtered through celite, and the solution was concentrated. After drying under vacuum, amino-terminated PDMS_0.37_-*co*-PDPS_0.63_ 6.0k was obtained with a yield of 0.99 g (87 %).

### 5.4. PDMS_0.37_-co-PDPS_0.63_ 6.0k-amic acid (Poly(amic acid) containing PDMS_0.37_-co-PDPS_0.63_ 6.0k)

Amino-terminated PDMS_0.37_-*co*-PDPS_0.63_ 6.0k 0.98 g (0.16 mmol) and THF (distilled and dried) 3 mL were added to a flask. To the solution, 4,4’-biphthalic anhydride (BPDA) 47 mg (0.16 mmol) and THF (distilled and dried) 1.9 mL were added. The solution was stirred for over 24 h under a reflux condition. After the reaction, the solution was reprecipitated into methanol, and the remaining solid was dried under a vacuum. PDMS_0.37_-*co*-PDPS_0.63_ 6.0k-amic acid was obtained with a yield of 0.48 g (47 %).

Figure S17. ^1^H-NMR spectrum of hydrosilyl-terminated PDMS_0.37_-*co-*PDPS_0.63_ 6.0k (solvent: CDCl_3_).

Figure S18. ^1^H-NMR spectrum of nitro-terminated PDMS_0.37_-*co-*PDPS_0.63_ 6.0k (solvent: CDCl_3_).

Figure S19. ^1^H-NMR spectrum of amino-terminated PDMS_0.37_-*co-*PDPS_0.63_ 6.0k (solvent: CDCl_3_).

Figure S20. ^1^H-NMR spectrum of PDMS_0.37_-*co-*PDPS_0.63_ 6.0k-amic acid (Solvent: THF-*d*_8_).

## Synthesis of PMPS_0.54_-*co*-PDPS_0.46_.4.1k

### 6.1. Hydrosilyl-terminated PMPS_0.54_-co-PDPS_0.46_.4.1k

1,3,5-Trimethyl-1,3,5-triphenylcyclotrisiloxane (D_3_^(Me,Ph)^) 1.5 g (3.7 mmol) and hexaphenylcyclotrisiloxane (D_3_^(Ph,Ph)^) 1.5 g (2.5 mmol) were dissolved in dried dichloromethane 1.0 mL. Into the solution, a mixture of H_2_O 18 mL (1.0 mmol) and dried THF 0.25 mL was added. A toluene solution of 1,3-trimethylene-2-propylguanidine (TMnPG) (91 mg / mL) 0.16 mL (containing 14 mg (0.10 mmol) of TMnPG) was added to initiate the polymerization. After 316 minutes of reaction at 30 ℃, chlorodimethylsilane 1.10 mL (10.0 mmol) and pyridine 1.31 mL (16.0 mmol) were added to terminate the reaction. The end-capping reaction was carried out for over 24 h. The mixture was concentrated and washed with acetonitrile. Drying under a vacuum afforded hydrosilyl-terminated PMPS_0.54_-*co*-PDPS_0.46_.4.1k with a yield of 2.26 g (75 %).

### 6.2. Nitro-terminated PMPS_0.54_-co-PDPS_0.46_.4.1k

Hydrosilyl-terminated PMPS_0.54_-*co*-PDPS_0.46_.4.1k 2.19 g (0.414 mmol), 1-(but-3-en-1-yloxy)-4-nitrobenzene 0.248 g (1.28 mmol), and dried toluene 10 mL were added to a test tube. After adding 16 drops of Karstedt’s cat., the reaction was carried out for over 24 h at 25 ℃. The progress of the reaction was verified by ^1^H-NMR of an aliquot of the reaction mixture. The mixture was concentrated and purified by column chromatography using hexane/dichloromethane (=2/1) as an eluent. Drying under a vacuum afforded nitro-terminated PMPS_0.54_-*co*-PDPS_0.46_.4.1k with a yield of 1.35 g (59 %).

### 6.3. Amino-terminated PMPS_0.54_-co-PDPS_0.46_.4.1k

Nitro-terminated PMPS_0.54_-*co*-PDPS_0.46_.4.1k 1.35 g, ethanol 4.6 mL, dichloromethane 4.5 mL, and Pd/C 0.1 g were added to a flask. The mixture was stirred at 30 ℃ under H_2_ atmosphere using H_2_ balloon. From the ^1^H-NMR spectrum of an aliquot of the reaction mixture, the complete conversion of nitro groups was verified. After the reaction, Pd/C was filtered through celite, and the solution was concentrated. After drying under vacuum, amino-terminated PMPS_0.54_-*co*-PDPS_0.46_.4.1k was obtained with a yield of 1.25 g (92 %).

### 6.4. PMPS_0.54_-co-PDPS_0.46_.4.1k-amic acid (Poly(amic acid) containing PMPS_0.54_-co-PDPS_0.46_.4.1k)

Amino-terminated PMPS_0.54_-*co*-PDPS_0.46_.4.1k 1.14 g (0.28 mmol) and THF (distilled and dried) 1.5 mL were added to a flask. To the solution, 4,4’-biphthalic anhydride (BPDA) 83 mg (0.28 mmol) and THF (distilled and dried) 1.5 mL were added. The solution was stirred for over 24 h under a reflux condition. After the reaction, the solution was reprecipitated into methanol, and the remaining solid was dried under a vacuum. PMPS_0.54_-*co*-PDPS_0.46_.4.1k-amic acid was obtained with a yield of 1.05 g (86 %).

Figure S22. ^1^H-NMR spectrum of nitro-terminated PMPS_0.54_-*co-*PDPS_0.46_ 4.1k (solvent: CDCl_3_).

Figure S21. ^1^H-NMR spectrum of hydrosilyl-terminated PMPS_0.54_-*co-*PDPS_0.46_ 4.1k (solvent: CDCl_3_).

Figure S23. ^1^H-NMR spectrum of amino-terminated PMPS_0.54_-*co-*PDPS_0.46_ 4.1k (solvent: CDCl_3_).

Figure S24. ^1^H-NMR spectrum of PMPS_0.54_-*co-*PDPS_0.46_ 4.1k-amic acid (solvent: THF-*d*_8_).

Figure S25. SEC chromatograms of hydrosilyl-terminated polysiloxanes. (Eluent: THF).


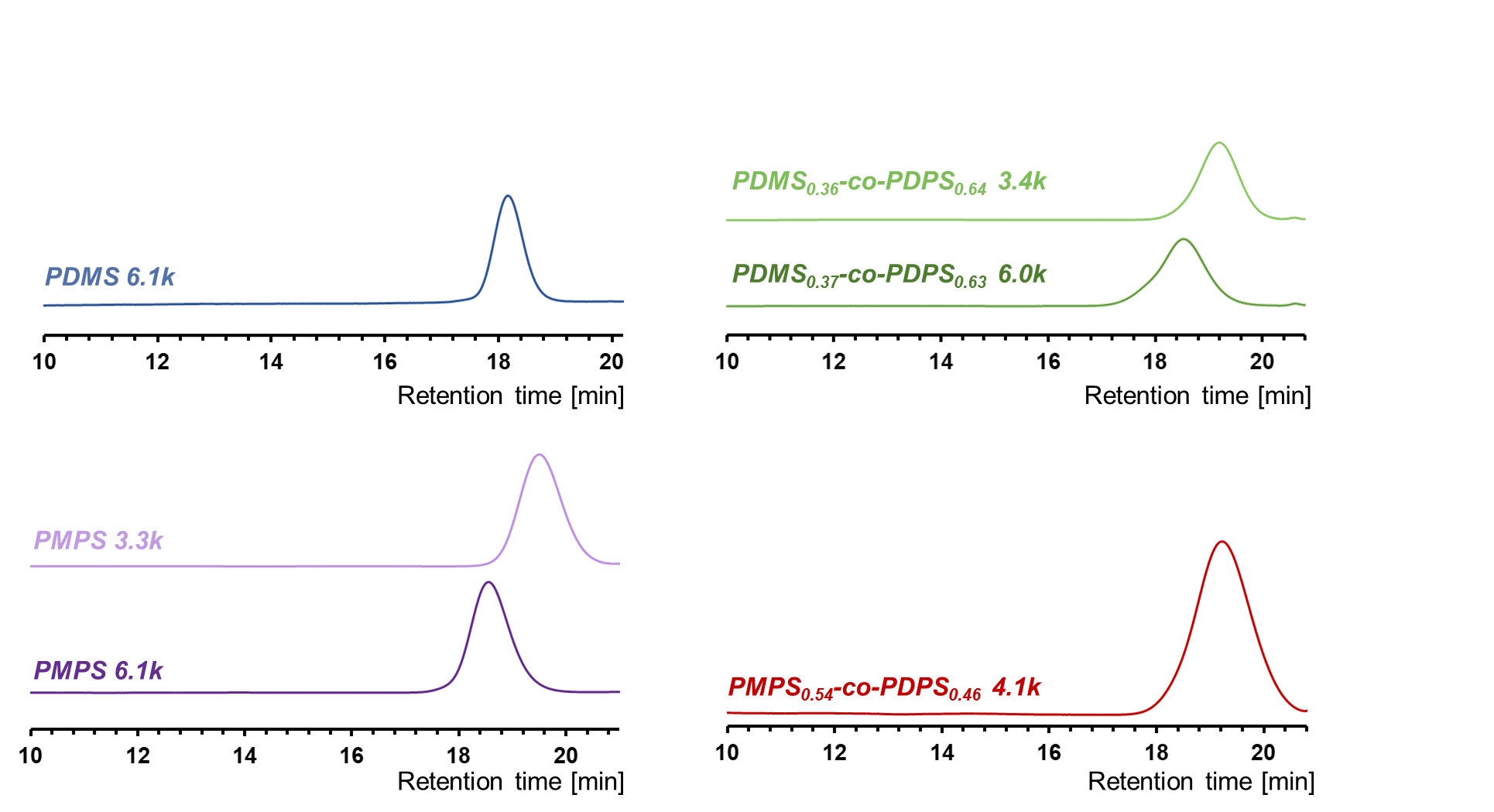


## Synthesis of polyimide composed of PMDA/ODA monomers.

PMDA (pyromellitic dianhydride)/ODA (4,4’-oxydianiline) polyimide was synthesized as follows. PMDA and dehydrated *N,N*-dimethylacetamide (DMAc) were added to a flask and the mixture was stirred to dissolve PMDA. Then, ODA and dehydrated DMAc were added to the solution. The reaction was conducted under nitrogen atmosphere. After 3 h, the solution was diluted and reprecipitated into methanol. PMDA/ODA poly(amic acid) was obtained as a yellowish solid after drying the residual solid at room temperature under vacuum. To prepare the polyimide, 10 wt.% DMAc solution of the poly(amic acid) was cast onto a glass substrate. After drying the sample, thermal treatment was conducted at 100 ℃ for 1 h, 200 ℃ for 1 h, and 300 ℃ for 2 h, affording the polyimide film.

# Thermal analyses.

## Glass transition temperatures estimated using Fox equation.

Calculated *T*_g_ values of copolymers using Fox equation are shown in Table S1.^1–3^

Table S1. Observed and calculated glass transition temperatures (*T*_g_) of amino-terminated polysiloxanes. Calculated values were estimated based on Fox equation using *T*_g_ value of -120 ℃ for PDMS and 40 ℃ for PDPS, according to the literature (ref. 1-3).

## TGA results of polysiloxanes.

Thermal stability of polysiloxanes measured by TGA are listed in Table S2. *T*_d5_ and *T*_d10_ represent the 5% and 10% weight loss temperatures, respectively. Hydrosilyl-terminated polysiloxanes with approximately the same molecular weight as those listed in Table 1 were used for this measurement.

Table S2. 5% and 10% weight loss temperature of hydrosilyl-terminated polysiloxanes upon heating under nitrogen flow.

# Dielectric properties of polysiloxane-imides.

## Relationship between dielectric loss tangent (*D*_f_) and glass transition temperature (*T*_g_).


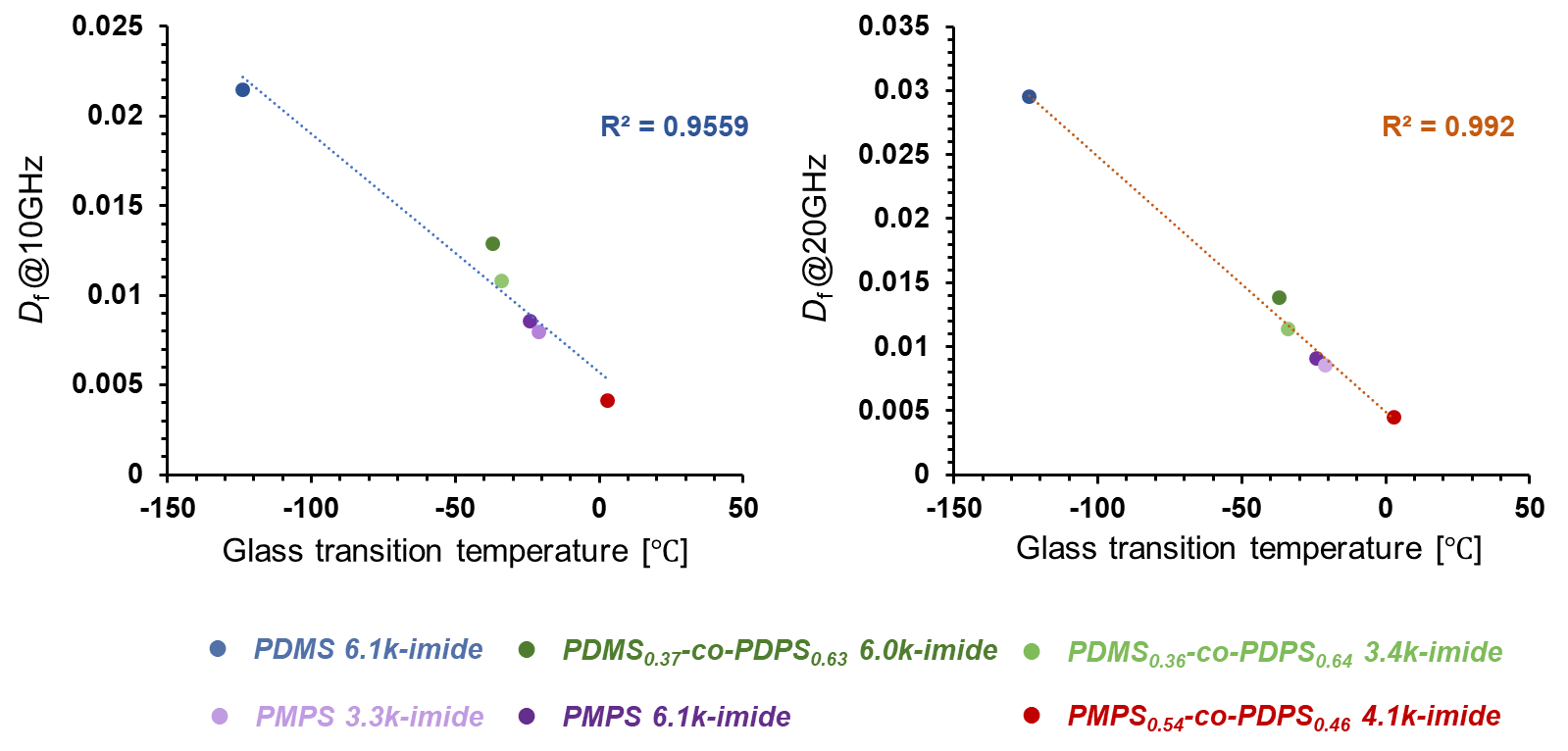


Figure S26. Relationship between *D*_f_ and *T*_g_ of polysiloxane-imide. Left: *D*_f_ at 10 GHz. Right: *D*_f_ at 20 GHz.

# WAXD analysis of polysiloxane-imides.

## The schematic image of polysiloxane-imide demonstrated by WAXD measurements.

Figure S27. Schematic image of polysiloxane-imides estimated from WAXD analyses.


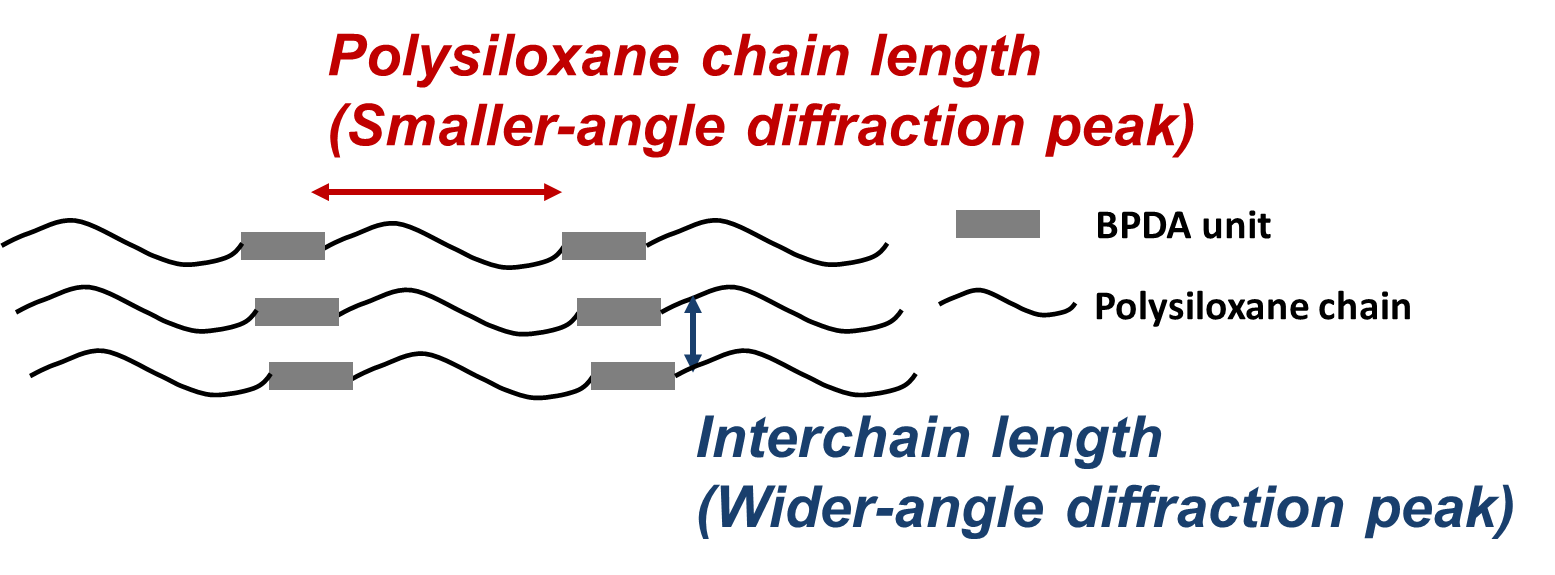


# Hydrophobicity evaluation of polysiloxane-imide films.

## Snapshots of contact angle measurements.

Figure S28. Snapshots of water droplets on the films of the typical polyimide and polysiloxane-imides.

# Calculation of molecular weight based on ^1^H-NMR spectrum.

The molecular weight estimated from ^1^H-NMR is calculated as follows, where *X*, *M*, and *I* are a degree of polymerization, molecular weight, and an integrated value of the corresponding peak. The subscript corresponds to the labels of each peak. For example, *I*_g_ for nitro-terminated PDMS means the integral of the peak g in Figure S2.

*Nitro-terminated PDMS*

$$X_{n,PDMS}={(1}/6)({4(I_{g}{+I}_{g^{'}})}/{I_{c}}-12)$$

$$M_{n,PDMS}=X_{n,PDMS}\times74.15+M_{n,terminal(NO2)}$$

$$M_{n,terminal\left( NO2 \right)}=520.73$$

*Nitro-terminated PMPS*

$$X_{n,PMPS}={(1}/3)({4\left( I_{g}+I_{k}-I_{\mathrm{TMS}} \right)}/{I_{c}}-12)$$

$$M_{n,PMPS}=X_{n,PMPS}\times136.23+M_{n,terminal(NO2)}$$

$$M_{n,terminal\left( NO2 \right)}=520.73$$

*Nitro-terminated PDMS-co-PDPS*

$$X_{n,PDMS}={(1}/6)({4{(I}_{g}+I_{g^{'}}+I_{h}-I_{\mathrm{TMS}})}/{I_{c}}-12)$$

$$X_{n,PDPS}=(1/10)({4{(I}_{b}+I_{i}{+I}_{j}+I_{k}-I_{\mathrm{Chloroform}})}/{I_{c}}-4)$$

$$M_{n,PDMS-co-PDPS}=X_{n,PDMS}\times74.15+{X_{n,PDPS}\times198.30+M}_{n,terminal(NO2)}$$

$$f_{\mathrm{PDMS}}=({X_{n,PDMS}\times74.15)}/{{(X}_{n,PDMS}\times74.15+X_{n,PDPS}\times198.30)}$$

$$M_{n,terminal(NO2)}=520.73$$

*Nitro-terminated PMPS-co-PDPS*

$$X_{n,PMPS}={(1}/3)({4{(I}_{g}+I_{g^{'}}+I_{k}-I_{\mathrm{TMS}})}/{I_{a}}-12)$$

$$X_{n,PDPS}=(1/10)({4{(I}_{b}+I_{h}{+I}_{i}{+I}_{j}+I_{l}{+I}_{m}{+I}_{n}-I_{\mathrm{Chloroform}})}/{I_{a}}-4-{5X}_{n,PMPS})$$

$$M_{n,PMPS-co-PDPS}=X_{n,PMPS}\times136.23+{X_{n,PDPS}\times198.30+M}_{n,terminal(NO2)}$$

$$f_{\mathrm{PMPS}}=({X_{n,PMPS}\times136.23)}/{{(X}_{n,PMPS}\times136.23+X_{n,PDPS}\times198.30)}$$

$$M_{n,terminal(NO2)}=520.73$$

# References.

(1) Wypych, G. PDMS Polydimethylsiloxane. In *Handbook of Polymers*; Wypych, G., Ed.; Elsevier: Oxford, 2012; pp 328–332. https://doi.org/10.1016/B978-1-895198-47-8.50102-8.

(2) Kalogeras, I. M.; Brostow, W. Glass Transition Temperatures in Binary Polymer Blends. *J Polym Sci B Polym Phys* **2009**, *47* (1), 80–95. https://doi.org/10.1002/polb.21616.

(3) Lee, M. K.; Meier, D. J. Synthesis and Properties of Diarylsiloxane and (Aryl/Methyl)Siloxane Polymers: 1. Thermal Properties. *Polymer* **1993**, *34* (23), 4882–4892. https://doi.org/10.1016/0032-3861(93)90013-Z.
